# The role of multimodality in clinical disease diagnosis: advances, challenges, and opportunities

**DOI:** 10.3389/fpubh.2026.1788454

**Published:** 2026-04-10

**Authors:** Asif Nawaz, Afaf Edinat, Muhammad Rizwan Rashid Rana, Tariq Ali, Ghulam Mustafa, Sidra Tahir, Seung Won Lee

**Affiliations:** 1 College of Information Technology, Amman Arab University, Amman, Jordan; 2 Department of Robotics & Artificial Intelligence, Shaheed Zulfikar Ali Bhutto Institute of Science and Technology, Islamabad, Pakistan; 3 University Institute of Information Technology, PMAS-Arid Agriculture University Rawalpindi, Rawalpindi, Pakistan; 4 Department of Precision Medicine, Sungkyunkwan University School of Medicine, Suwon, Republic of Korea; 5 Department of Artificial Intelligence, Sungkyunkwan University, Suwon, Republic of Korea; 6 Department of Metabiohealth, Sungkyunkwan University, Suwon, Republic of Korea; 7 Personalized Cancer Immunotherapy Research Center, Sungkyunkwan University School of Medicine, Suwon, Republic of Korea; 8 Department of Family Medicine, Kangbuk Samsung Hospital, Sungkyunkwan University School of Medicine, Seoul, Republic of Korea

**Keywords:** clinical decision support, data fusion, deep learning, disease diagnosis, medical imaging, multimodal learning

## Abstract

Advances in artificial intelligence (AI) have significantly improved medical diagnosis, with deep learning models achieving expert-level performance across unimodal tasks such as medical imaging, physiological signal analysis, electronic health record (EHR) modeling, and omics-based prediction. However, clinical decision-making is inherently multimodal, as diseases manifest through complex interactions among imaging phenotypes, molecular signatures, physiological measurements, and textual clinical documentation. Consequently, unimodal systems often lack robustness, generalizability, and clinical reliability. This survey provides a comprehensive and methodologically grounded review of multimodal learning for disease diagnosis, emphasizing the paradigm shifts that have emerged over the past five years. Beyond classical early, intermediate, and late fusion strategies, we synthesize modern cross-modal representation learning frameworks, including contrastive alignment, vision–language pretraining, graph and hypergraph-based multimodal reasoning, modality-agnostic representation learning, and missing-modality robust architectures. We further examine large-scale foundation-model style multimodal pretraining and recent advances in histology–transcriptomics and image–omics integration, which exemplify biologically grounded cross-modal learning beyond traditional fusion pipelines. In addition to summarizing widely used datasets and clinical applications across oncology, neurology, cardiology, pulmonology, and ophthalmology, we provide a methodological synthesis linking key challenges such as modality heterogeneity, incomplete data, fairness disparities, interpretability limitations, and cross-institutional distribution shift to representative solution frameworks proposed in the literature. By integrating theoretical formulations, architectural insights, and application-driven evidence, this survey moves beyond case-oriented performance comparisons and offers a structured perspective on how multimodal AI is evolving toward scalable, robust, and clinically trustworthy diagnostic systems.

## 1 Introduction

**Figure 1:**
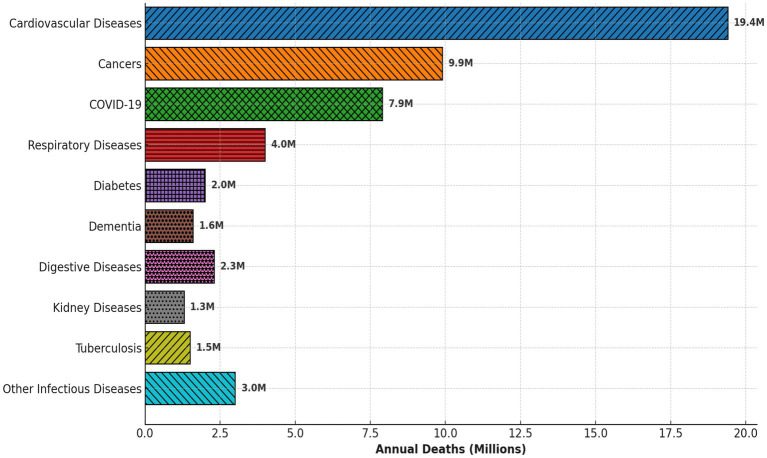
Global causes of death 148.

Diseases, both communicable and non-communicable, continue to pose a significant burden on global health, impacting millions of lives every year as presented in Figure 1 (1, 2). Infectious diseases, such as COVID-19, HIV/AIDS, and malaria, have devastating effects on public health, particularly in low-income regions where access to healthcare resources is limited. At the same time, chronic non-communicable diseases (NCDs) like cardiovascular diseases, diabetes, cancer, and neurological disorders have emerged as the leading cause of mortality and morbidity worldwide. According to the World Health Organization (WHO), NCDs account for nearly 70% of global deaths, with a growing prevalence due to aging populations, lifestyle changes, and environmental factors (3). These diseases not only cause suffering for individuals but also place a tremendous strain on healthcare systems, economies, and societies (4). The economic burden of disease includes direct medical costs, loss of productivity, and long-term care requirements, which can be crippling for both individuals and countries. Furthermore, the rising incidence of multi-morbidity, where individuals suffer from multiple diseases simultaneously, adds another layer of complexity to disease management, making timely and accurate diagnosis essential for effective treatment and prevention strategies (5).

Clinical decision-making in healthcare involves several distinct but interconnected tasks, including screening, diagnosis, and triage. Screening typically focuses on identifying individuals at risk of disease in large populations, often using rapid or low-cost tests such as chest X-rays for tuberculosis or mammography for breast cancer (6). Diagnosis, on the other hand, is the process of establishing the presence or absence of disease in symptomatic individuals, often requiring integration of multiple forms of evidence such as imaging, laboratory tests, and clinical history (7). Triage involves prioritizing patients based on the urgency of their condition, as in emergency departments where rapid assessment determines immediate care pathways. While these tasks overlap, the accuracy and timeliness of diagnosis remain central to patient outcomes, influencing both treatment effectiveness and long-term prognosis.

In this context, multimodality the integration of heterogeneous data sources such as medical images, clinical notes, structured electronic health records (EHR), laboratory tests, and even wearable or genomic data has emerged as a transformative paradigm. Each modality captures unique aspects of disease biology and patient health: imaging provides anatomical or functional insights, laboratory results quantify biochemical changes, and clinical text captures nuanced physician interpretations (8). Relying on a single modality risk overlooking critical information, whereas combining complementary signals enhances robustness, reduces the chance of diagnostic error, and can help mitigate bias by ensuring that predictions are not disproportionately dependent on a single data type (9). Furthermore, multimodal systems have shown promise in delivering fairer and more equitable care, as they can incorporate broader patient contexts, reducing disparities that often arise when models are trained on limited or homogeneous data (10–12).

The scope of this survey is deliberately focused on multimodal methods for clinical diagnosis. We review research published over the past five years that integrates at least two modalities to support diagnostic decision-making in human healthcare. Studies that focus solely on prognosis, treatment response prediction, or risk stratification are excluded unless they explicitly include diagnostic endpoints. Similarly, we exclude purely synthetic or preclinical studies unless their findings are directly applicable to clinical diagnostic tasks. Our goal is to systematically synthesize how multimodal approaches have been applied across diseases, to evaluate their added diagnostic value, and to identify the methodological advances, challenges, and future opportunities in this rapidly evolving domain.

Although several surveys have explored multimodal learning for medical applications, most of them concentrate on specific tasks such as medical image analysis or multimodal data fusion techniques. This review differs from existing literature by providing a systematic and cross-disciplinary perspective on multimodal disease diagnosis, integrating insights from imaging, clinical records, physiological signals, and molecular data. Furthermore, this survey goes beyond traditional fusion taxonomies by examining emerging representation learning paradigms, including cross-modal contrastive learning, multimodal foundation models, and graph-based reasoning frameworks. By combining methodological analysis with application-oriented evidence across multiple disease domains, this work aims to bridge the gap between algorithmic innovation and clinical diagnostic practice.

### 1.1 Research contributions

This survey makes the following key contributions:

- We systematically trace the progression from unimodal diagnostic models (imaging, signals, EHR, omics) to multimodal integration, presenting a clear *road* map of modality with structured comparisons.
- This work provides a detailed analysis of fusion methods (early, intermediate, and late fusion) with diagrams, equations, and representative studies, highlighting how methodological advances translate into improved diagnostic outcomes.
- Consolidate the most widely used multimodal medical datasets and evaluation benchmarks, while also summarizing domain-specific applications in oncology, neurology, cardiology, ophthalmology, and beyond.
- To critically examine the limitations such as missing modalities, data imbalance, interpretability, and fairness, and outline open problems and future directions for the development of clinically deployable multimodal AI systems.

## 2 Scope of the survey

The purpose of this survey is to provide a comprehensive overview of the role and importance of multimodal data in disease diagnosis, with a particular focus on developments over the past five years. The survey is motivated by the rapid growth of research in medical AI that moves beyond unimodal approaches toward more integrated, clinically robust multimodal frameworks. Our aim is not only to summarize existing literature but also to critically analyze the progress, limitations, and emerging trends that define the field. The scope of this review is restricted to clinical diagnosis tasks that involve identifying the presence, type, or stage of a disease. Specifically, we include works that integrate two or more modalities such as medical imaging (e.g., Magnetic Resonance Imaging (MRI), Computed Tomography (CT), X-rays, pathology slides), physiological signals [e.g., Electrocardiogram (ECG), Electroencephalogram (EEG)], structured clinical data [e.g., laboratory tests, vitals, Electronic Health Records (EHR)], textual data (e.g., clinical notes, radiology reports), and molecular/omics data (e.g., genomics, transcriptomics, proteomics). Studies that focus exclusively on unimodal methods are mentioned only as baselines to provide context for the need for multimodality. By contrast, we exclude literature that primarily addresses prognostic modeling (e.g., survival analysis, treatment outcome prediction) or non-diagnostic clinical tasks such as resource optimization, workflow automation, or administrative applications, unless the studies also incorporate diagnostic endpoints. Furthermore, works that explore multimodality in non-clinical settings, such as general medical Natural Language Processing (NLP) or healthcare operations research, are outside the scope of this survey. Finally, this survey is application-oriented: we emphasize disease domains such as oncology, neurology, cardiology, pulmonology, and ophthalmology, where multimodal approaches have demonstrated measurable improvements over unimodal baselines. In addition, we highlight the methodological innovations in fusion strategies, benchmark datasets, and evaluation metrics that are shaping the future of multimodal diagnostic AI.

## 3 Background and definitions

To fully understand the value of multimodal data in disease diagnosis, it is important to define what is meant by *modality* in the clinical context. A modality refers to a distinct source or type of medical information that captures different aspects of a patient’s health. For a given patient p, we can represent their health record as a collection of modalities. For a given patient, we can represent their health record as a collection of modalities, as defined in Equation 1:


$$M(p)=\{m_{1}\;(p),m_{2}\;(p),\ldots ,m_{k}\;(p)\}$$ (1)

Where $m_{i}(p)$ denotes the data from the $i^{\mathrm{th}}$ modality (e.g., CT scan, lab results, ECG waveform). The diagnostic function f maps this multimodal input to a predicted diagnosis $y^{\prime }$ as formulated in Equation 2:


$$y^{\prime }=\{m_{1}\;(p),m_{2}\;(p),\ldots ,m_{k}\;(p)\}$$ (2)

Where, $y^{\prime }\in Y$ the set of possible diagnostic labels.

A unimodal approach uses only one $m_{i}(p)$ , whereas multimodal approaches exploit the joint distribution of modalities as given in Equation 3:


$$P(y|m_{1},m_{2},\ldots ,m_{k})\neq \prod_{i=1}^{k}P(y|m_{i})$$ (3)

Demonstrating that diagnostic accuracy often arises from the *complementarity* of signals, not from their isolated contributions.

### 3.1 Taxonomy of modalities

**Figure 2:**
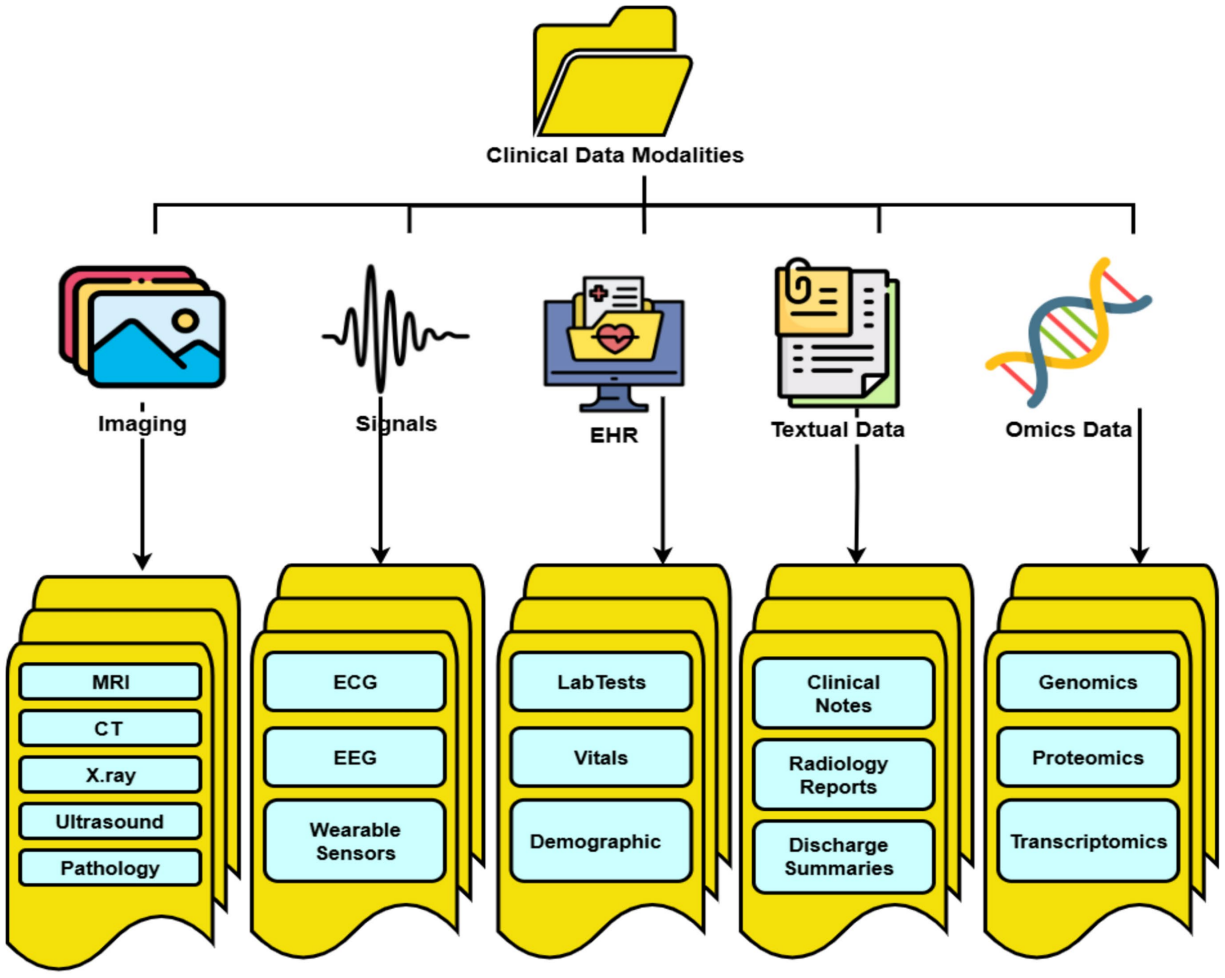
Taxonomy of clinical data modalities.

A fundamental step in understanding multimodal disease diagnosis is to clearly classify the types of data modalities available in clinical practice. The taxonomy of modalities as presented in Figure 2, spans from imaging-based data such as MRI, CT, and X-rays, to non-imaging signals like ECG and EEG, as well as structured electronic health records, textual clinical notes, and molecular omics data.

This categorization provides the foundation for analyzing how different modalities can be integrated to enhance diagnostic performance.

- Medical imaging ( $m_{\mathrm{img}}$ ): Radiology scans (X-ray, CT, MRI, PET), ultrasound, pathology slides, dermatology photographs, and ophthalmology OCT. Imaging provides spatial and morphological features as expressed in Equation 4:

$$m_{\mathrm{img}}\in {\mathbb{R}}^{H\times W\times C}$$ (4)

Where H, W, C denote image dimensions and channels.

- Clinical text $\left(m_{\text{text}}\;\right)$ : Physician notes, radiology reports and discharge summaries. Represented as token sequences as given in Equation 5:

$$m_{\text{text}}=\{w_{1},w_{2},\ldots ,w_{T}\},\;\;w_{t}\in V$$ (5)

where V is the vocabulary set.

- Structured EHR data $\left(m_{\mathrm{ehr}}\;\right)$ : Tabular features such as labs, vitals and demographics as formulated in Equation 6:

$$m_{\mathrm{ehr}}\in R^{d}$$ (6)

with d being the number of clinical variables.

- Physiological signals $\left(m_{\text{signal}}\;\right)$ : Time-series like ECG or EEG, typically modeled as Equation 7:

$$m_{\text{signal}}=\{s_{1},s_{2},\ldots ,s_{T}\},\;\;s_{t}\in R^{n}$$ (7)

where *n* is the number of channels.

- Omics data ( $m_{\text{omics}}$ ): High-dimensional molecular profiles: as expressed in Equation 8:

$$m_{\text{omics}}\in R^{g}$$ (8)

where g is the number of genes or molecular features.

- Wearables & IoT $\left(m_{\text{wear}}\;\right)$ : Continuous monitoring signals such as heart rate, activity, or glucose levels, often represented as multi-resolution time-series.

### 3.2 Diagnosis vs. prognosis

In clinical practice, it is essential to distinguish between diagnosis, which focuses on detecting and classifying a disease at a given point in time, and prognosis, which predicts the likely future course or outcome of a disease. While prognosis is important for treatment planning, this survey primarily emphasizes diagnostic tasks, since timely and accurate diagnosis forms the cornerstone of effective patient management and is the main area where multimodal data integration has shown the greatest impact.

Formally, diagnosis seeks to estimate Equation 9:


$$y_{\mathrm{diag}}^{\prime }=f_{\theta }\;(M(p)),\;\;\;\;y_{\mathrm{diag}}^{\prime }\in Y_{\text{disease}}\;$$ (9)

Whereas prognosis models disease evolution over time Equation 10:


$$y_{\text{prog}}^{\prime }\;(t)=g_{\phi }\;(M(p),t),\;\;\;y_{\text{prog}}^{\prime }\in Y_{\text{outcomes}}$$ (10)

This survey focuses on diagnostic tasks, i.e., estimating $y_{\mathrm{diag}}^{\prime }$ , Prognostic models are only included if they explicitly incorporate diagnostic endpoints.

## 4 Methodology

**Figure 3:**
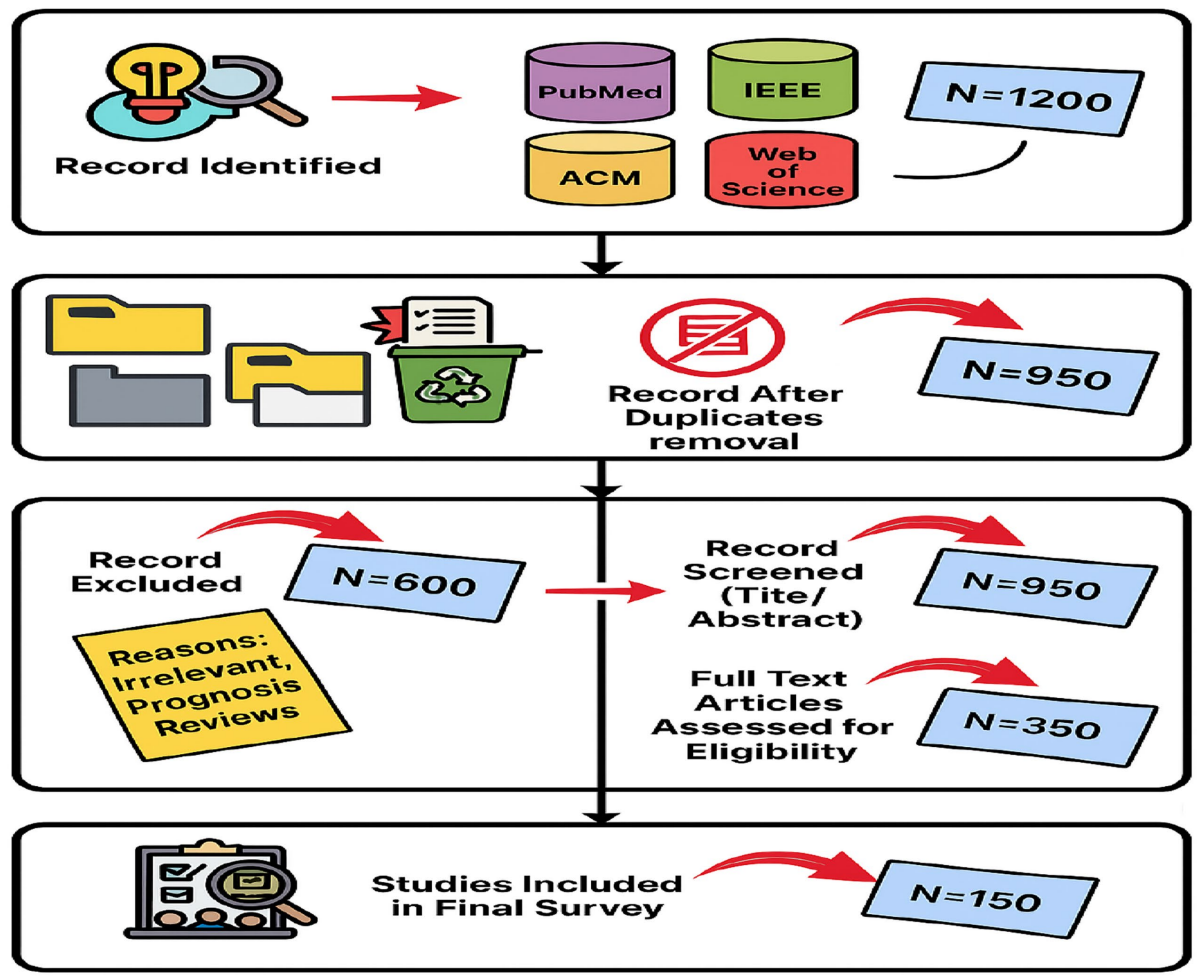
Literature search flow of the proposed work based on PRISMA-style.

To ensure a comprehensive and reproducible survey, we adopted a systematic review approach inspired by Preferred Reporting Items for Systematic Reviews and Meta-Analyses (PRISMA) guidelines as presented in Figure 3. Our methodology consists of three stages: literature search, study selection, and data extraction/analysis.

### 4.1 Literature search strategy

**Figure 4:**
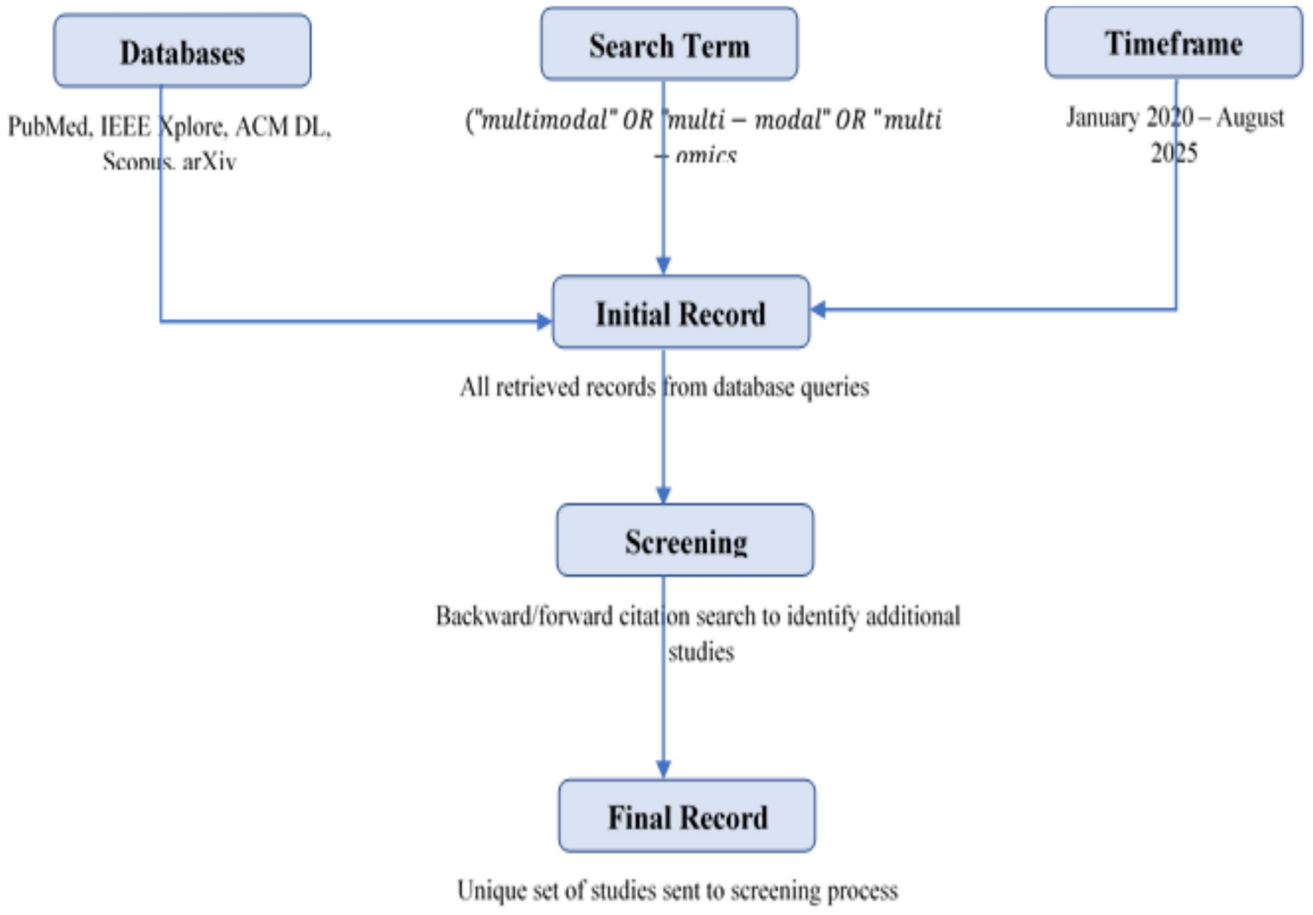
Literature search strategy.

We searched multiple scientific databases including PubMed, IEEE Xplore, ACM Digital Library, Scopus, and arXiv for papers published between January 2020 and August 2025. Search queries combined disease diagnosis keywords with multimodality terms. Figure 4 shows the workout flow of literature search study also showing the example search string as:


("multimodal"OR"multi−modal"OR"multi−omics")

$$\text{AND}\;(\begin{array}{l} "\text{disease diagnosi}s"\;OR"\text{clinical diagnosi}s"\;\\ \text{OR}"\text{medical diagnosi}s" \end{array})$$

We also screened references of included papers to identify additional relevant studies.

### 4.2 Inclusion and exclusion criteria

To ensure that only high-quality and relevant studies were considered, we defined strict inclusion and exclusion criteria as presented in Table 1, prior to the screening process. Studies were included if they (i) were published between 2020 and 2025, (ii) focused on human disease diagnosis and utilized two or more modalities, (iii) reported quantitative diagnostic performance metrics (e.g., Area Under the Receiver Operating Characteristic Curve (AUROC), Area Under the Precision–Recall Curve (AUPRC), accuracy, or F1-score), and (iv) provided a clear comparison with unimodal or ablation baselines.

**Table 1:** The criteria for the selection of relevant material.

Inclusion criteria	Exclusion criteria
Published between 2020–2025	Published before 2020 or after 2025
Focus on human disease diagnosis	Focus only on prognosis, risk prediction, or treatment response
Use of two or more modalities (e.g., imaging + EHR, pathology + genomics)	Single-modality studies without multimodal integration
Report quantitative diagnostic performance metrics (AUROC, AUPRC, F1-score, etc.)	No diagnostic performance reporting or only qualitative findings
Include comparison with unimodal or ablation baselines	Case reports or very small sample size studies (*N* < 30)
Peer-reviewed or widely cited preprints	Opinion pieces, editorials, reviews, or purely methodological works without experiments

In contrast, studies were excluded if they focused solely on prognosis, treatment response, or risk prediction without diagnostic endpoints, or if they were based on synthetic/non-clinical datasets. Case reports and studies with insufficient sample size (e.g., fewer than 30 patients) were also excluded, as were purely methodological papers, review articles, or opinion pieces that lacked experimental evaluation. This careful filtering ensured that the final pool of studies directly addressed the role of multimodal data in improving diagnostic accuracy and were suitable for synthesis in this survey.

## 5 Road map of modality

The trajectory of artificial intelligence in medical diagnosis can be understood as a progressive road map of modality. In the early stages, most research relied on unimodal approaches, where a model is trained on a single data type such as imaging, physiological signals, clinical records, or omics data. In such cases, the predictive function can be represented as Equation 11:


$$\hat{y}=f(m),P(y|m)$$ (11)

Where mmm denotes a single modality and f(·) is a modality-specific function, often a convolutional neural network for imaging, a recurrent network for physiological signals, or a sequence model for EHR. These unimodal approaches have achieved remarkable breakthroughs, demonstrating that deep learning can rival or even surpass human experts in certain constrained domains. For instance, convolutional neural networks trained on retinal fundus images can detect diabetic retinopathy at ophthalmologist-level performance, while dermatology models can classify skin cancer with accuracy comparable to board-certified specialists. Similar progress has been reported in chest X-ray interpretation, where models such as CheXNet achieved radiologist-level accuracy in pneumonia detection, and in computational pathology, where weakly supervised networks have been able to identify cancer on whole-slide images without detailed annotations. In the domain of physiological signals, deep neural networks trained on ECGs have achieved cardiologist-level accuracy in arrhythmia classification, while EEG-based deep learning models have advanced seizure detection. Electronic health records have also been successfully modeled using deep sequence architectures, enabling the prediction of mortality, hospital readmission, and length-of-stay. Finally, omics data such as genomics and transcriptomics have proven highly effective in tumor classification and tissue-of-origin prediction, with deep neural networks extracting clinically meaningful patterns from vast molecular datasets. Table 2 provides a consolidated summary of representative unimodal literature across imaging, physiological signals, EHR, and omics, demonstrating the milestones achieved in single-modality diagnosis.

**Table 2:** Representative unimodal diagnostic literature.

Modality	References	Disease/task	Key findings
Imaging	Gulshan et al. (36), Esteva et al. (37), Rajpurkar et al. (38), Campanella et al. (39), and Oakden-Rayner et al. (40)	DR, skin cancer, pneumonia, pathology cancer detection, chest X-ray	Achieved expert-level or near-expert performance in specific image-based tasks. Limitations: modality-specific, lacks context from other data.
Physiological signals	Hannun et al. (41), Acharya et al. (42), Yildirim et al. (43), Faust et al. (44), and Song et al. (45)	Arrhythmia, seizure detection, sleep apnea, stress detection	High accuracy from raw signals, outperforming hand-crafted features. Limitations: modality alone insufficient for systemic disease diagnosis.
EHR & clinical notes	Rajkomar et al. (46), Miotto et al. (47), Shickel et al. (48), Huang et al. (49), and Solares et al. (50)	Clinical prediction, readmission, mortality	EHR-based models predict multiple outcomes at scale. Limitations: missing structural/biological signals.
Genomics & omics	Jiao et al. (51), Way & Greene. (52), Khodabakhshi et al. (53), Chen et al. (54), and Divate et al. (55)	Tumor classification, pan-cancer analysis	Omics-based models classify cancers with high accuracy. Limitations: costly data, missing phenotypic context.

Despite their impressive achievements, unimodal approaches face inherent limitations. A single modality, by its very nature, captures only one aspect of disease. For instance, an MRI scan may reveal structural brain changes, but it cannot capture molecular mechanisms driving those changes. Conversely, genomics may reveal mutational patterns but lacks the phenotypic context necessary for clinical interpretation. This leads to blind spots that reduce the robustness and generalizability of unimodal models. The shortcomings of unimodal analysis can be formalized through probability theory, where the assumption that the joint conditional probability can be decomposed as Equation 12:


$$P!(y|m_{1},m_{2},\ldots ,m_{k})=\prod_{i=1}^{k}P!(y|m_{i})$$ (12)

is almost never valid in clinical practice. In reality, as expressed in Equation 13:


$$P!(y|m_{1},m_{2},\ldots ,m_{k})\neq \prod_{i=1}^{k}P!(y|m_{i})$$ (13)

because different modalities capture complementary, interdependent aspects of the disease process. This inequality highlights the central motivation for multimodality: integrating multiple sources of information is not just additive but synergistic, offering diagnostic insights that no single modality can provide.

The importance of multimodality lies in three key principles. First, it enables complementarity, as each modality contributes distinct and non-redundant information for example, PET scans measure metabolism while MRI captures structure, and together they offer a more comprehensive view of neurodegeneration. Second, multimodality ensures robustness, allowing one modality to compensate for noise or missingness in another, a common occurrence in real-world clinical practice. Third, multimodality promotes fairness and generalizability, as relying on diverse data reduces the risk of overfitting to biases in any single modality. Collectively, these factors explain why multimodal fusion consistently outperforms unimodal baselines in disease diagnosis. The general formulation of multimodal models involves learning modality-specific representations through functions $f_{i}(m_{i})$ and then combining them via a fusion function F(·) , expressed as Equation 14:


$$\hat{y}=F!(f_{1}(m_{1}),f_{2}(m_{2}),\ldots ,f_{k}(m_{k}))$$ (14)

Where fusion strategies may include simple concatenation, attention mechanisms, cross-modal transformers, or graph-based embeddings. Such models operationalize the theoretical advantage of joint learning by enabling rich interactions across heterogeneous modalities.

Table 3 highlights representative multimodal successes in disease diagnosis. For example, integrating histopathology and genomics has yielded improved cancer subtype classification and survival prediction compared to either modality alone. Similarly, Alzheimer’s disease diagnosis has advanced through the joint analysis of MRI, PET, and clinical data, allowing earlier and more accurate detection. In pulmonary disease triage, combining chest X-rays with EHR data through multimodal transformers has enhanced ICU risk prediction and outperform unimodal systems. Ophthalmology has benefited from integrating fundus and OCT imaging, improving sensitivity to subtle manifestations of diabetic retinopathy and glaucoma. In cardiovascular medicine, combining ECG signals with laboratory tests and EHR features has led to more accurate prediction of cardiac risk and mortality. Taken together, this road map of modality demonstrates the natural progression of diagnostic AI. Unimodal models establish strong baselines and validate the utility of deep learning in medical tasks, but their inherent limitations underscore the need for multimodal fusion. By integrating structural, temporal, clinical, and molecular evidence, multimodal models consistently achieve superior accuracy, robustness, and fairness. They move diagnosis closer to the way clinicians reason in practice—by synthesizing diverse pieces of evidence into a coherent decision. Thus, the success of multimodality represents not just an incremental step but a paradigm shift in disease diagnosis, laying the groundwork for the fusion architectures that will be discussed in the following section.

**Table 3:** Representative multimodal success stories.

Modality combination	References	Disease/task	Key findings
Histopathology + Genomics	Chen et al. (17), Couture et al. (56), Wang et al. (57), Hao et al. (58), and Sun et al. (59)	Cancer subtype classification, prognosis	Improved survival prediction & subtype classification vs. imaging or omics alone
MRI + PET + Clinical data	Suk et al. (60), Liu et al. (61), Zhang et al. (62), Qiu et al. (63), and Li et al. (64)	Alzheimer’s disease diagnosis	Earlier and more accurate AD detection than unimodal MRI or PET
CXR + clinical notes (EHR)	Zhang et al. (65) Johnson et al. (66) Huang et al. (67), Wu et al. (68), and Tang et al. (69)	Pulmonary disease detection, ICU triage	Outperformed radiology-only or EHR-only baselines in chest disease detection
Fundus + OCT imaging	Ting et al. (70), Chakravarty et al. (71), Liu et al. (72), Bhardwaj et al. (31), and Jiang et al. (73)	Diabetic retinopathy, glaucoma	Improved detection of subtle ophthalmic changes using multimodal vision
ECG + EHR/labs	Harutyunyan et al. (74), Shashikumar et al. (75), Choi et al. (76), Reyna et al. (77), and Zhao et al. (78)	Cardiovascular risk stratification, mortality	Boosted predictive performance for cardiac outcomes and ICU survival

## 6 Datasets and benchmarks

The development of multimodal diagnostic models has been strongly influenced by the availability of large-scale datasets that integrate diverse data types as presented in Table 4. Datasets such as MIMIC-CXR + MIMIC-IV and CheXpert provide imaging data linked with radiology reports and EHR information, enabling studies on chest disease diagnosis and automated report generation. In oncology, The Cancer Genome Atlas (TCGA) and Clinical Proteomic Tumor Analysis Consortium (CPTAC) stand out as rich resources that combine histopathology images with genomics, transcriptomics, and proteomics, thus supporting research on precision cancer diagnostics. Similarly, Alzheimer’s Disease Neuroimaging Initiative (ADNI) has facilitated advances in neurodegenerative disease diagnosis by providing Magnetic Resonance Imaging (MRI), Positron Emission Tomography (PET) imaging, cognitive scores, and genetic data. These resources allow researchers to move beyond unimodal analysis and explore how complementary information across imaging, text, omics, and structured EHR can improve diagnostic accuracy and robustness.

**Table 4:** Representative multimodal datasets for disease diagnosis.

Dataset	Modalities	Disease area/use case	Scale
MIMIC-CXR + MIMIC-IV	X-ray, radiology reports, EHR	Chest disease diagnosis, report generation	370 k + patients
CheXpert	Chest X-ray, reports	Thoracic disease classification	224 k images
ADNI	MRI, PET, cognitive scores, genetics	Alzheimer’s, MCI vs. healthy controls	1,700 + subjects
TCGA	Pathology WSIs, genomics, transcriptomics	Cancer diagnosis and subtyping	11,000 + patients
CPTAC	Histology, proteomics, genomics	Cancer proteogenomics	10 + tumor types
MIMIC-III/IV	EHR, clinical notes, ECG, labs	Critical care diagnostics	60 k ICU stays
PhysioNet (various)	ECG, EEG, PPG, PCG	Cardiac, neurological, sleep disorder diagnosis	Varies
UK biobank	Fundus/OCT imaging, EHR	Ophthalmic disease (e.g., DR, AMD)	500 k participants
ISIC archive	Dermoscopy + metadata	Skin cancer diagnosis	70 k + images

Despite these advances, multimodal datasets remain unevenly distributed across medical domains. Areas such as dermatology and ophthalmology benefit from resources like ISIC and UK Biobank, but many other diseases particularly in low-resource and rare disease settings lack publicly available multimodal data. Moreover, even well-established datasets often suffer from limitations such as class imbalance, missing modalities, and lack of external validation across diverse patient populations. These challenges underscore the need for new benchmarks that are representative, diverse, and clinically realistic, as well as standardized evaluation protocols to fairly assess multimodal methods. Without these, progress in multimodal diagnostic research risks being siloed and difficult to translate into real-world clinical practice.

Not all datasets labeled as “multimodal” provide the same level of integration or diagnostic utility. Truly multimodal datasets as presented in Table 5, such as MIMIC-CXR + MIMIC-IV, ADNI, TCGA, and CPTAC, contain complementary data sources (e.g., imaging, omics, EHR, or cognitive assessments) that enable direct study of cross-modal interactions and fusion strategies. These resources allow researchers to investigate how different data types jointly contribute to diagnostic accuracy. By contrast, enriched-unimodal datasets, such as CheXpert, PhysioNet, and the ISIC Archive, primarily consist of a single dominant modality (e.g., chest X-rays or dermoscopic images) with limited metadata or textual labels attached. While these datasets remain valuable for certain multimodal tasks such as image-to-text learning or metadata conditioning they do not capture the full spectrum of multimodal integration that is essential for real-world diagnosis. Recognizing this distinction is crucial, as it prevents overestimating the diversity of available data and highlights the pressing need for richer, more clinically representative multimodal benchmarks.

**Table 5:** Multimodal vs. enriched-unimodal datasets for disease diagnosis.

Dataset	Modalities included	Type	Notes
MIMIC-CXR + MIMIC-IV	Chest X-ray + Radiology Reports + Structured EHR	Multimodal	Strongly linked imaging–text–EHR; benchmark for multimodal fusion
CheXpert	Chest X-ray + Radiology Reports	Multimodal (limited)	Text-image pairs, but no structured EHR or labs
ADNI	MRI + PET + Cognitive Scores + Genetics	Multimodal	Rich for neurodegenerative disease diagnosis
TCGA	Histopathology WSIs + Genomics + Transcriptomics	Multimodal	Gold-standard for cancer omics + pathology
CPTAC	Histology + Proteomics + Genomics	Multimodal	Extends TCGA with proteogenomics
MIMIC-III/IV	Structured EHR + Clinical Notes + Labs + ECG Signals	Multimodal	Covers ICU patients, widely used for diagnostic AI
PhysioNet (various)	ECG/EEG/PCG/PPG + Some metadata	Primarily Unimodal	Often signal-only datasets, though sometimes paired with demographic data
UK biobank	Fundus/OCT Imaging + HER	Multimodal	Very large, supports ophthalmic studies
ISIC archive	Dermoscopic Images + Patient Metadata	Enriched-Unimodal	Primarily image-based; metadata is sparse

Despite the progress enabled by existing resources, there remain critical gaps in multimodal datasets for disease diagnosis. Current benchmarks are heavily concentrated on common adult diseases in high-income regions, leaving pediatric and rare disease populations underrepresented. This imbalance limits the ability of multimodal models to generalize to vulnerable groups where diagnostic uncertainty is often greatest. Similarly, most datasets originate from well-resourced healthcare systems, highlighting the need for multimodal resources in low- and middle-income countries to ensure diagnostic AI tools are equitable and globally relevant. Another key limitation is that many datasets are cross-sectional snapshots, whereas real-world diagnosis often unfolds across time; future benchmarks should therefore integrate longitudinal data combining imaging, EHR, and wearable streams to capture disease progression. In addition, truly robust multimodal datasets must be collected across multiple centers and diverse populations to reduce bias and enhance fairness. Finally, the field would benefit from not just more datasets, but also from standardized benchmarking protocols with defined train/test splits and baseline models, enabling fair comparison and accelerating progress toward clinically reliable multimodal diagnostics.

## 7 Modeling and fusion architectures

**Figure 5:**
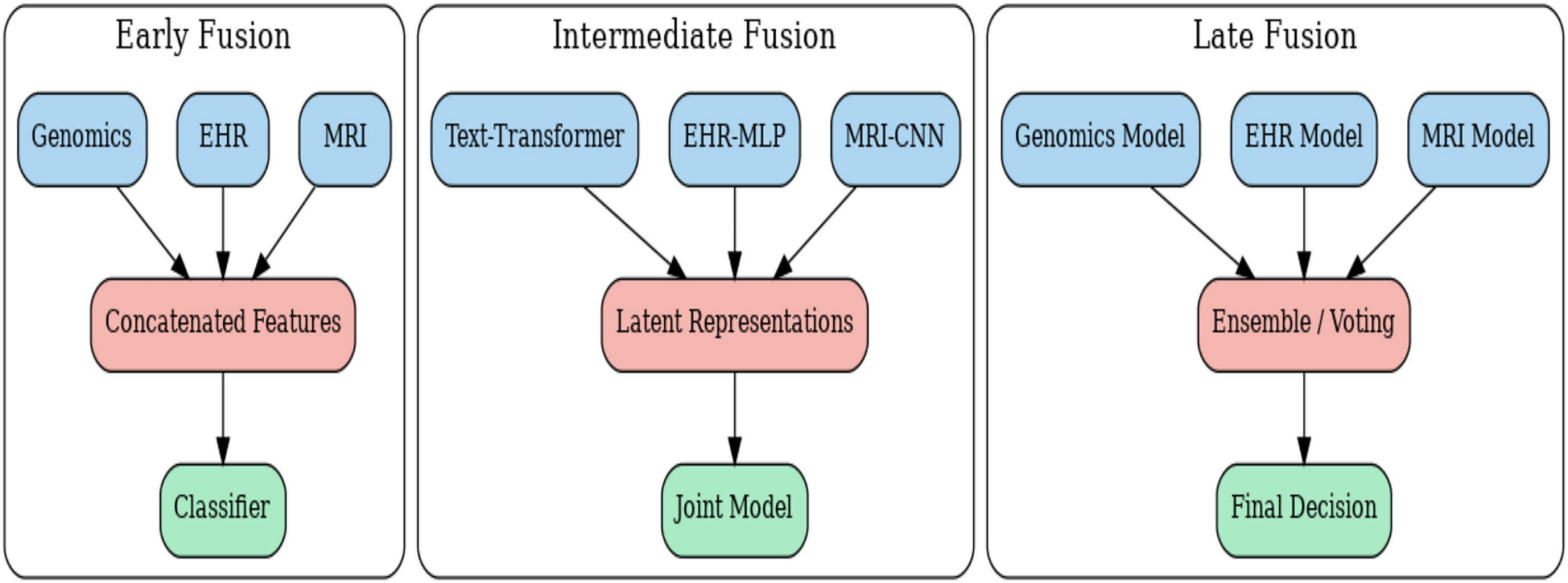
Fusion strategies for multimodal disease diagnosis.

A central challenge in multimodal disease diagnosis lies in determining how to integrate heterogeneous data sources into a unified predictive framework. Fusion strategies are typically categorized into three main approaches as presented in Figure 5: early fusion, intermediate fusion, and late fusion. Each approach makes different assumptions about how modalities interact, and each comes with unique strengths and limitations.

### 7.1 Early fusion (feature-level integration)

Early fusion involves concatenating or combining features extracted from multiple modalities before passing them into a classifier. Formally, if $z_{i}=h_{i}(m_{i})$ represents the learned representation of modality $m_{i}$ , the fused vector is obtained as in Equation 15:


$$z_{\text{fusion}}={⊕}_{i=1}^{k}z_{i}$$ (15)

Where ⊕ denotes concatenation. A classifier f then maps $z_{\text{fusion}}$ to the final prediction. This approach is straightforward and effective when modalities are well-aligned, such as fusing chest X-rays with corresponding radiology reports. However, it often ignores higher-order cross-modal dependencies and is highly sensitive to missing data.

### 7.2 Intermediate fusion (joint representation learning)

Intermediate fusion aims to capture cross-modal interactions during the feature learning process. Instead of concatenation, specialized architectures such as transformers, attention mechanisms, or co-embedding networks are employed. For example, in a cross-attention setting, one modality $\left(z_{a}\right)$ can be refined by attending to another $\left(z_{b}\;\right)$ it is given in Equation 16:


$$z_{a}^{\prime }=\text{Attention}(Q=z_{a},K=z_{b},V=z_{b})$$ (16)

This strategy allows the model to highlight complementary information across modalities, such as aligning genomic features with histopathology patterns. Intermediate fusion has been shown to improve diagnostic robustness and accuracy but requires significant computational resources and careful handling of alignment across heterogeneous data types.

### 7.3 Late fusion (decision-level integration)

Late fusion combines decisions rather than raw features. Each modality-specific model outputs a probability distribution $p_{i}(y)$ over diagnostic classes. These are then aggregated to form the final decision as given in Equation 17:


$$\hat{y}=\arg\max_{y}\sum_{i=1}^{k}\alpha_{i}\;p_{i}(y)$$ (17)

where $\alpha_{i}$ are modality-specific weights. This strategy is attractive for its flexibility it is robust to missing modalities and allows modular integration of independently trained unimodal models. However, because it only aggregates predictions, it often fails to exploit fine-grained interdependencies between modalities. The Table 6 summarizes the three main fusion paradigms in multimodal disease diagnosis.

**Table 6:** Comparative analysis of fusion strategies.

Fusion strategy	Mathematical formulation	Advantages	Limitations
Early fusion (feature-level)	$z_{i}=h_{i}(m_{i}),i=1,\ldots ,k$ $z_{\text{fusion}}={⊕}_{i=1}^{k}z_{i}$ $\hat{y}=f(z_{\text{fusion}})$	Simple, effective when data is well-aligned.	Ignores complex dependencies; sensitive to missing modalities.
Intermediate fusion (joint learning)	$z_{\text{fusion}}=g(z_{1},z_{2},\ldots ,z_{k})$ $z_{a}^{\prime }=\text{Attention}(Q=z_{a},K=z_{b},V=z_{b})$	Captures cross-modal dependencies; robust.	Computationally expensive; alignment challenges.
Late fusion (decision-level)	$p_{i}=f_{i}(m_{i}),i=1,\ldots ,k$ $\hat{y}=\arg\max_{y}\sum_{i=1}^{k}\alpha_{i}\;p_{i}(y)$	Flexible, robust to missing modalities.	Loses fine-grained cross-modal correlations.

To illustrate how fusion strategies have been applied in practice, Table 7 summarizes representative studies from the past five years that applied multimodal fusion for disease diagnosis. Each entry highlights the fusion methodology employed (early, intermediate, or late fusion variants), the disease domain, and the corresponding reference. As shown, oncology and neurology have led much of the methodological innovation, often employing intermediate fusion through attention-based models or tensor fusion to integrate imaging with omics or neuroimaging with clinical measures. Cardiology and pulmonology applications frequently use early or intermediate strategies to combine physiological signals, imaging, and EHR data, while ophthalmology and dermatology have seen more recent adoption of attention-based image–metadata fusion.

**Table 7:** Representative literature in multimodal fusion in disease diagnosis.

References	Fusion methodology	Disease type
Chen et al. (79)	Intermediate fusion (pathology WSI + multi-omics; tensor/attention style)	Oncology (multi-cancer)
Chen et al. (17)	Intermediate fusion (tensor fusion + gated attention)	Oncology
Tang et al. (18)	Intermediate fusion (cross-attention/dual transformer)	Neurology (Alzheimer’s)
Duong et al. (80)	Middle/intermediate fusion (cross-attention)	Neurology (early AD)
Jahanian et al. (20)	Intermediate fusion (vision–language transformer)	Pulmonology/Thoracic imaging
Soto et al. (19)	Intermediate fusion (joint modeling of ECG & echo time-series)	Cardiology (LVH/HCM)
Subhalakshmi et al. (81)	Early/feature-level fusion (deep features + classifier)	Pulmonology (COVID-19)
Tur et al. (82)	Early fusion (tabular biomarkers + image embeddings)	Pulmonology (COVID-19)
El-Ateif et al. (24)	Early vs. intermediate vs. late (comparative)	Ophthalmology
Ou et al. (83)	Intermediate fusion (attention-based image–metadata fusion)	Dermatology (skin cancer)
Veluchamy et al. (84)	Intermediate fusion (multi-task learning)	Pulmonology (COVID-19)

The table demonstrates that fusion strategy selection often depends on modality characteristics: early fusion is commonly used when modalities are structurally similar (e.g., tabular biomarkers and imaging embeddings), intermediate fusion is preferred when modeling complex cross-modal dependencies (e.g., histopathology and genomics), and late fusion is favored in high-stakes diagnostic settings where robustness to missing modalities is critical. Overall, this comparative evidence reinforces the importance of aligning fusion methodology with clinical context and available data modalities.

## 8 Modern paradigms in multimodal learning

Multimodal learning has undergone substantial conceptual evolution over the past few years. While early, intermediate, and late fusion strategies provided an important architectural foundation for integrating heterogeneous clinical data, recent advances have shifted the field toward representation-centric and large-scale pretraining paradigms. Rather than focusing primarily on how to combine modality-specific features, contemporary approaches emphasize learning aligned, transferable patient representations across heterogeneous data streams. This transition reflects broader developments in machine learning, including self-supervised learning, contrastive representation alignment, graph-based reasoning, and foundation-model pretraining. In the context of disease diagnosis, these paradigms aim not only to improve predictive accuracy but also to enhance robustness, generalization across institutions, and adaptability under missing-modality scenarios. The following subsections synthesize these modern methodological directions and their implications for multimodal diagnostic AI.

### 8.1 Cross-modal contrastive learning

In recent years, cross-modal contrastive learning has emerged as one of the most influential paradigms in multimodal artificial intelligence, fundamentally reshaping how heterogeneous medical data are integrated. Unlike classical fusion approaches that explicitly concatenate or jointly encode modality-specific features, contrastive frameworks aim to learn aligned representation spaces by maximizing agreement between semantically paired modalities while minimizing similarity to unrelated pairs. Formally, given two modalities $m_{a}$ and $m_{b}$ , with encoder functions $f_{a}(\cdot )$ and $f_{b}(\cdot )$ , the resulting embeddings are are given in Equation 18:


$$z_{a}=f_{a}(m_{a}),z_{b}=f_{b}(m_{b})\;$$ (18)

Contrastive learning optimizes an objective such as the InfoNCE loss as given in Equation 19:


$${ℒ}_{\text{contrastive}}=-\log\frac{\exp(\mathrm{sim})(z_{a},z_{b})/\tau )}{\sum_{k=1}^{N}\exp(\mathrm{sim})(z_{a},z_{b})/\tau )}$$ (19)

Where sim(·) denotes cosine similarity and τ is a temperature parameter. This objective encourages aligned multimodal representations without requiring explicit feature concatenation or handcrafted fusion rules.

The key conceptual advance of cross-modal contrastive learning lies in reframing multimodality as a representation alignment problem rather than a fusion engineering task. Instead of asking how to combine modalities, contrastive learning asks how to make different modalities represent the same patient state in a shared latent space. This paradigm offers several advantages such as reduced dependence on labeled data through, self-supervised pretraining, improved cross-domain transferability, nnatural robustness to partial modality availability and scalable foundation-model style pretraining. In medical AI, contrastive frameworks have been applied across imaging–text, imaging–EHR, pathology–omics, and signal–tabular integrations. Table 8 summarizes recent representative studies so far applying cross-modal contrastive learning to disease diagnosis and clinical prediction tasks.

**Table 8:** Cross-modal contrastive learning in multimodal disease diagnosis.

Study	Modalities	Contrastive strategy	Clinical task	Dataset
Ding et al. (85)	Medical imaging + structured data	Cross-graph modal contrastive learning	Image classification	Clinical dermatology datasets
Li et al. (86)	Histopathology + genomics	Cross-modal contrastive + attention alignment	Survival prediction	TCGA
Jing et al. (8)	Multi-modal MRI/CT	Hypergraph contrastive learning	Image segmentation	BraTS, lung CT datasets
Gu et al. (88)	Imaging + tabular clinical data	Contrastive multimodal fusion with modality dropout	Disease detection	Multi-center hospital datasets
Pan et al. (89)	Radiology images + reports	Self-supervised image–text contrastive pretraining	Radiology diagnosis	Large uncurated clinical datasets
Liu et al. (90)	Medical image + expert annotations	Enhanced CLIP-style contrastive learning (eCLIP)	Multimodal classification	Multi-institution datasets

### 8.2 Vision–language pretraining and representation alignment

Building upon contrastive learning principles, vision–language pretraining has emerged as a dominant paradigm in multimodal biomedical AI. Inspired by large-scale models such as CLIP, this approach leverages paired image–text corpora to learn semantically aligned embeddings through self-supervised objectives. In medical contexts, these corpora typically consist of radiological images and reports, pathology slides and molecular descriptions, or imaging studies paired with structured clinical narratives. Rather than designing explicit fusion modules, vision–language frameworks as presented in Table 9, jointly train modality-specific encoders to project heterogeneous inputs into a shared embedding space in which semantically corresponding image–text pairs are closely aligned.

**Table 9:** Vision–language pretraining and representation alignment in multimodal disease diagnosis.

Study	Modalities	Pretraining strategy	Clinical application	Dataset
Zhang et al. (91)	Chest X-ray + Radiology reports	Self-supervised contrastive vision–language pretraining	Thoracic disease classification	MIMIC-CXR
Boecking et al. (92)	Medical images + Clinical text	Semantic-aware vision–language alignment	Radiology understanding tasks	Multi-institution datasets
Li et al. (93)	Imaging + Reports	Contrastive language–image pretraining	Diagnostic classification	Chest X-ray datasets
Wang et al. (94)	Medical images + Text (unpaired)	Contrastive pretraining with unpaired data	Cross-domain classification	Multiple public datasets
Azizi et al. (95)	Medical images + Textual supervision	Large-scale self-supervised pretraining	General medical image classification	Multi-modal biomedical datasets
Huang et al. (96)	Imaging + EHR + Clinical notes	Multimodal deep representation alignment	Clinical outcome prediction	Hospital EHR-linked imaging datasets

The core objective typically combines contrastive alignment with masked modeling or cross-attention mechanisms. Given an image embedding $z_{\mathrm{img}}$ and a text embedding $z_{\text{text}}$ , alignment is enforced through similarity maximization, while transformer-based cross-attention layers may further refine modality interactions. Unlike classical early or intermediate fusion strategies that require tight architectural coupling, vision–language pretraining enables flexible downstream adaptation, allowing models to be fine-tuned for classification, report generation, retrieval, or zero-shot diagnostic inference. In radiology, large-scale pretraining on chest X-ray and report pairs has demonstrated that aligned representations capture clinically meaningful associations between visual abnormalities and textual descriptions. These models have shown improved transfer performance across institutions and diagnostic tasks compared to unimodal imaging models trained from scratch. Similar approaches in pathology integrate whole-slide images with molecular or diagnostic descriptors, enabling cross-modal reasoning in oncology. Beyond imaging, vision–language techniques have also been extended to align dermatological images with patient metadata and ophthalmic scans with clinical annotations, improving both predictive accuracy and semantic interpretability.

A defining characteristic of vision–language pretraining is its scalability. By exploiting vast archives of uncurated clinical data, these models learn generalized biomedical representations that reduce reliance on manually annotated labels. This property is particularly valuable in public health settings where large-scale labeled datasets may be unavailable. Furthermore, aligned multimodal embeddings support retrieval-based decision support systems, enabling clinicians to query similar prior cases across modalities. However, despite these advantages, several challenges remain. Large-scale paired medical datasets are often institution-specific and difficult to aggregate due to privacy constraints. Alignment learned through contrastive objectives may capture spurious correlations present in clinical documentation practices rather than underlying pathophysiology. Additionally, interpretability remains an open concern, as alignment scores do not inherently reveal causal relationships between modalities. Computational demands during large-scale pretraining further limit accessibility in resource-constrained environments. Nevertheless, vision–language pretraining represents a substantial conceptual advancement beyond classical fusion paradigms. By reframing multimodal integration as a representation alignment problem grounded in large-scale self-supervision, this approach aligns with the broader trajectory toward foundation-model development in healthcare AI. In the context of disease diagnosis, such models hold promise for improving robustness, generalization, and scalability, thereby contributing to more equitable and deployable multimodal diagnostic systems.

In addition to dual-encoder contrastive alignment, recent vision–language frameworks employ cross-attentional multimodal transformers that enable bidirectional interaction between visual tokens and textual tokens. Given image feature tokens $X\in {\mathbb{R}}^{n\times d}$ and report tokens $T\in {\mathbb{R}}^{m\times d}\;$ cross-attention is computed as in Equation 20:


$$\begin{array}{l} \text{Attention}(Q=XW_{Q},K=TW_{K},V=TW_{V})\\ =\text{softmax}(\frac{QK^{T}}{\sqrt{d}})V \end{array}$$ (20)

allowing textual clinical descriptions to guide spatial refinement of image representations. Such report-guided modeling enhances disease localization and semantic grounding in radiology. Moreover, multimodal pretraining increasingly integrates masked language modeling (MLM), masked image modeling (MIM), and contrastive objectives within a unified loss formulation in Equation 21:


$$ℒ={ℒ}_{\mathrm{MLM}}+{ℒ}_{\mathrm{MIM}}+{ℒ}_{\text{contrastive}}$$ (21)

enabling large-scale self-supervised learning from uncurated hospital archives. Recent multimodal medical foundation models extend this paradigm by incorporating structured EHR variables, clinical notes, and imaging features within unified transformer architectures, facilitating cross-modal reasoning across heterogeneous patient records. These large-scale pretraining strategies have demonstrated improved zero-shot transfer, cross-institutional generalization, and robustness compared to task-specific fusion pipelines, marking a decisive methodological shift toward scalable multimodal representation learning in clinical AI.

### 8.3 Graph-based and hypergraph multimodal reasoning

Graph-based multimodal reasoning represents a structurally distinct paradigm for integrating heterogeneous clinical data. Unlike contrastive alignment, which emphasizes representation similarity across modalities, graph-based frameworks explicitly model relationships among modalities, features, and patients as structured relational systems. This approach is particularly well suited to healthcare settings, where biological processes, clinical variables, and imaging findings are inherently interconnected rather than independent signals requiring simple fusion.

In graph-based multimodal models as presented in Table 10, heterogeneous data sources are represented as nodes within a graph G = (V, E), where V denotes the set of nodes and E the set of edges encoding relationships among modalities or features. Each node $v_{i}\in V$ may correspond to imaging features, genomic markers, laboratory variables, or patient-level representations. Edges encode relational dependencies such as biological pathways, clinical co-occurrence patterns, or learned similarity structures. Graph neural networks (GNNs) update node representations through message passing mechanisms. A typical layer-wise propagation rule is expressed as in Equation 22:


$$h_{i}^{\left(l+1\right)}=\sigma (W^{\left(l\right)}\sum_{j\in N(i)}\frac{1}{c_{\mathrm{ij}}}h_{j}^{\left(l\right)}$$ (22)

**Table 10:** Graph-based multimodal reasoning in disease diagnosis.

Study	Modalities	Graph strategy	Clinical task	Dataset
Li et al. (97)	Imaging	Graph Neural Network (GNN) survey framework	Medical image analysis	Multiple datasets
Zhou et al. (98)	Imaging + Genomics	Multimodal GNN	Cancer survival prediction	TCGA
Wang et al. (99)	Multi-imaging modalities	Hypergraph neural network	Medical image segmentation	Neuroimaging datasets
Gao et al. (100)	Imaging + HER	Multimodal graph learning	Disease diagnosis	Hospital datasets
Fu et al. (101)	Histopathology + Genomics	Graph-based multimodal fusion	Tumor subtype classification	Oncology cohorts
Zhang et al. (102)	EHR + Imaging	Graph attention network	Clinical risk prediction	Multi-center datasets

Where $h_{j}^{\left(l\right)}$ denotes the representation of node iii at layer l, N(i) represents neighboring nodes, $c_{\mathrm{ij}}$ is a normalization factor, $W^{\left(l\right)}$ is a learnable weight matrix, and σ(·) is a non-linear activation function. Through iterative message passing, node embeddings capture higher-order dependencies across modalities. Hypergraph extensions further generalize this framework by allowing hyperedges to connect more than two nodes simultaneously, enabling modeling of multi-way interactions. The hypergraph convolution can be formulated as in Equation 23:


$$H^{\left(l+1\right)}=\sigma (D_{v}^{-\frac{1}{2}}HW_{\epsilon }{D_{\epsilon }}^{-1}D_{v}^{-\frac{1}{2}}H^{\left(l\right)}W^{\left(l\right)}),$$ (23)

Where, H denotes the incidence matrix, $D_{v}$ ​ and $D_{e}$ ​are degree matrices for vertices and hyperedges, and $W_{e}$ ​ encodes hyperedge weights. Such formulations allow integration of complex relationships among imaging, molecular, and clinical modalities beyond pairwise fusion.

Graph-based multimodal reasoning offers several conceptual advantages over classical fusion paradigms. By explicitly encoding structural relationships among modalities, it captures interdependencies that may reflect biological causality rather than simple feature co-occurrence. Graph attention mechanisms further enhance interpretability by revealing which modality interactions contribute most to diagnostic decisions. Moreover, relational modeling can improve generalization by leveraging similarity structures across patient cohorts, mitigating overfitting to site-specific patterns.

However, graph-based methods also introduce challenges. Constructing clinically meaningful graphs requires domain knowledge or robust similarity estimation, and graph architectures may be computationally intensive for large-scale datasets. Additionally, interpretability, while improved relative to black-box fusion, depends on the reliability of learned graph attention weights. Despite these limitations, graph and hypergraph-based multimodal reasoning represents a major methodological evolution beyond early, intermediate, and late fusion taxonomies, aligning multimodal disease diagnosis with structured relational learning principles.

### 8.4 Modality-agnostic representation learning

A further conceptual advancement in multimodal learning is the emergence of modality-agnostic representation learning, which seeks to construct unified patient embeddings that are invariant to the specific modality through which information is observed. Unlike classical fusion strategies that require modality-specific encoders followed by explicit integration mechanisms, modality-agnostic frameworks as presented in Table 11, aim to learn a shared latent space in which heterogeneous inputs imaging, text, structured clinical data, signals, or omics are projected into a common representation domain. In this setting, the emphasis shifts from combining modality-dependent features to learning modality-invariant patient descriptors.

**Table 11:** Representative studies on modality-agnostic.

Study	Modalities	Representation strategy	Dataset/domain
Baltrušaitis et al. (103)	General multimodal	Unified multimodal representation taxonomy	Cross-domain
Tsai et al. (104)	Language + multimodal sequences	Multimodal transformer (unaligned sequences)	Multimodal datasets
Huang et al. (105)	Imaging + EHR	Deep shared latent representations	Hospital datasets
Rajkomar et al. (93)	EHR + structured signals	Scalable unified patient embeddings	Large-scale EHR
Solares et al. (46)	EHR modalities	Deep neural shared encoders	EHR systems
Wu et al. (106)	Imaging + tabular data	Domain-invariant multimodal representation	Clinical datasets
Zhou et al. (107)	Multi-imaging + clinical data	Modality-invariant embedding learning	Medical imaging cohorts
Zhang et al. (108)	EHR + imaging	Unified multimodal representation framework	Multi-center hospital datasets
Ngiam et al. (109)	Audio + vision (general ML)	Shared deep multimodal latent space	Benchmark datasets
Ganin and Lempitsky (110)	Cross-domain data	Adversarial domain-invariant learning	General ML

Formally, given heterogeneous modalities $\left\{m_{1},m_{2},\ldots ,m_{k}\right\}$ , modality-agnostic learning defines encoders $f_{i}(\cdot )$ that map each modality into a shared latent space Z expressed in Equation 24:


$$z_{i}=f_{i}(m_{i}),z_{i}\in Z$$ (24)

The objective is to minimize modality-specific discrepancies while preserving patient-level semantics. This can be expressed as in Equation 25:


$$ℒ={ℒ}_{\text{task}}+\lambda_{\text{Linvariance}}$$ (25)

Where ${ℒ}_{\text{task}}$ ​ denotes the primary diagnostic objective (e.g., classification loss) and $\lambda_{\text{Linvariance}}$ enforces alignment across modalities, often through adversarial training or distribution matching. In adversarial modality-invariant learning, a discriminator D(·) attempts to identify the originating modality of a latent representation, while the encoders are trained to prevent such discrimination formulated in Equation 26:


$$.f_{i}\min\mathrm{ED}\max[\log D(z_{i}\;)]+E[\log(1-D(z_{j}\;))]$$ (26)

Through this minimax objective, the learned representations become increasingly indistinguishable across modalities, encouraging a unified embedding space. In clinical contexts, modality-agnostic learning is particularly valuable because real-world patient records are inherently incomplete and heterogeneous. A patient may have imaging data but limited laboratory tests, or rich EHR data without genomic sequencing. By constructing modality-invariant representations, models can generalize across varying input configurations and maintain consistent predictive behavior even when specific modalities are absent.

Recent studies have applied modality-agnostic principles in imaging–EHR integration, where patient-level embeddings are learned to be robust across structured and unstructured data streams. Similarly, in oncology, latent embeddings derived from histopathology and genomics have been constrained to occupy shared representation spaces, enabling downstream survival prediction independent of modality-specific distortions. In multimodal ICU risk prediction, unified embeddings have allowed dynamic fusion of time-series vitals and clinical notes without requiring explicit modality-specific pipelines. These approaches align with the broader movement toward foundation-style biomedical models that treat multimodal data as interchangeable manifestations of an underlying patient state. Compared to contrastive learning, which emphasizes pairwise alignment between modalities, modality-agnostic representation learning prioritizes global invariance across all modalities simultaneously. Compared to graph-based reasoning, which models explicit structural relationships, modality-agnostic approaches abstract away modality identity altogether. This abstraction offers improved flexibility and scalability, particularly when new modalities are introduced, as the shared latent space can accommodate additional encoders without redesigning fusion mechanisms.

However, the modality-agnostic paradigm introduces its own challenges. Enforcing invariance may inadvertently suppress clinically meaningful modality-specific signals, particularly when certain modalities contain unique diagnostic information not present elsewhere. Moreover, adversarial objectives can be unstable during training and require careful balancing between task performance and invariance regularization. Interpretability also becomes more complex, as unified embeddings may obscure the contribution of individual modalities to diagnostic outcomes. Despite these limitations, modality-agnostic representation learning represents a significant evolution beyond classical fusion taxonomies. By reframing multimodal diagnosis as the estimation of a unified latent patient state, this paradigm aligns with the broader objective of precision medicine: integrating diverse biomedical signals into coherent, transferable representations capable of supporting robust and equitable clinical decision-making across heterogeneous healthcare environments.

### 8.5 Missing-modality robust architectures

A persistent challenge in real-world multimodal healthcare systems is incomplete modality availability. Unlike curated research datasets, clinical practice rarely provides a fully observed multimodal profile for every patient. Imaging may be available without genomic data, laboratory values may be missing, or textual reports may be incomplete. Classical fusion architectures, particularly early and intermediate fusion models, typically assume full modality presence and therefore degrade substantially when inputs are absent. This limitation has motivated the development of missing-modality robust architectures that explicitly model partial observations. Table 12 presents some of the core architecture develop so far.

**Table 12:** Missing-modality robust architectures in multimodal disease diagnosis.

Study	Modalities	Robustness strategy	Dataset/domain
Ma et al. (111)	Vision + text	Transformer masking for missing modalities	Vision-language datasets
Hazarika et al. (112)	Multimodal signals	Modality-invariant + modality-specific decomposition	Multimodal benchmarks
Wu et al. (113)	Imaging + tabular	Domain-invariant learning	Clinical datasets
Zhou et al. (108)	Multi-imaging + clinical	Modality-invariant embedding learning	Imaging cohorts
Han et al. (109)	Imaging + structured data	Gated mixture-of-experts	Medical datasets
Nguyen et al. (114)	Multimodal data	Joint representation learning	Benchmark datasets
Tran et al. (115)	Multimodal signals	GAN-based modality imputation	Benchmark datasets
Rahman et al. (116)	Text + vision	Large transformer with partial masking	Large corpora
Li et al. (117)	Imaging + EHR	Uncertainty-aware multimodal fusion	Hospital datasets
Parthasarathy and Busso (118)	Multimodal signals	Multi-task learning under missing signals	Signal datasets

Formally, consider a multimodal input set $M(p)=\{m_{1},m_{2},\ldots ,m_{k}\}$ for patient p. In practice, only a subset $M_{\mathrm{obs}}(p)\subseteq M(p)$ is observed. The predictive objective becomes is given in Equation 27:


$$y^{\prime }=f(M_{\mathrm{obs}}(p))$$ (27)

Where the model must remain stable under arbitrary modality subsets. Robust multimodal architectures therefore aim to learn functions invariant to modality dropout Equation 28:


$$f(M_{\mathrm{obs}}(p))\approx f(M(p))\;\forall M_{\mathrm{obs}}\subseteq M$$ (28)

One widely adopted strategy is modality dropout during training, where modalities are randomly masked to simulate real-world missingness. This forces the network to distribute predictive capacity across modalities rather than relying disproportionately on a single dominant source. The objective can be expressed as Equation 29:


$$ℒ=E_{S∼P(M)}[\ell (f(M_{S}),y)]\;$$ (29)

Where S represents sampled modality subsets.

Another approach employs mixture-of-experts (MoE) architectures, where modality-specific experts $f_{i}(m_{i})$ are combined via a gating network g(·) Equation 30:


$$y^{\prime }=\sum_{i=1}^{k}g_{i}(M_{\mathrm{obs}})\;f_{i}(m_{i})\;$$ (30)

Here, the gating mechanism dynamically adjusts modality weights depending on availability, naturally accommodating partial inputs. Generative approaches have also been proposed, in which missing modalities are reconstructed using conditional generative models it is expressed in Equation 31:


$$m_{j}^{\prime }=G(m_{i}),i\neq j$$ (31)

Allowing imputation of absent modalities prior to fusion. More recent transformer-based architectures incorporate modality-aware masking tokens that permit attention mechanisms to operate across incomplete sequences without structural redesign.

In oncology, missing-modality strategies have been applied to histopathology–genomics integration, where genomic sequencing may not be available for all patients. In critical care settings, ICU risk prediction models must accommodate incomplete laboratory panels and intermittent physiological signals. Multimodal radiology systems frequently encounter absent or sparsely documented reports. Studies incorporating modality dropout and MoE-based gating have demonstrated improved robustness under simulated missingness compared to rigid fusion architectures. Similarly, uncertainty-aware fusion models quantify predictive confidence when modalities are incomplete, supporting safer deployment in clinical decision-making. Despite these advances, missing-modality learning remains an open challenge. Training models under all possible modality subsets becomes combinatorically complex as the number of modalities increases. Generative reconstruction risks propagating noise or synthetic artifacts. Furthermore, robustness to missingness does not guarantee fairness; if certain populations systematically lack specific modalities, models may inadvertently encode structural disparities. Consequently, future research must integrate robustness, uncertainty quantification, and fairness-aware modeling within missing-modality frameworks.

## 9 Applications across disease domains

**Figure 6:**
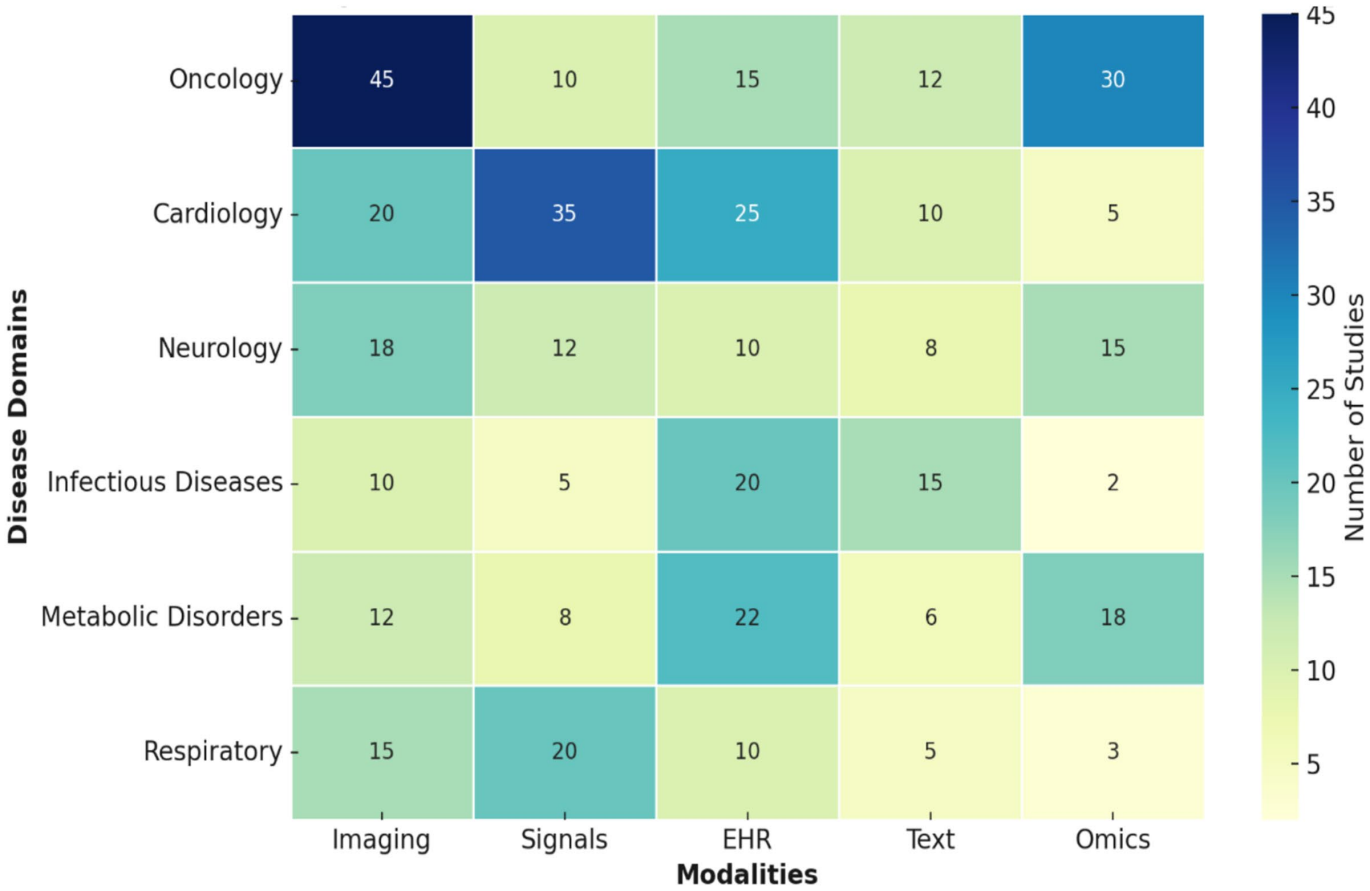
Heatmap of modalities used across disease domains (illustrative).

Multimodal learning has been applied across diverse medical domains, each leveraging different combinations of data sources to enhance diagnostic performance. Figure 6 shows the heatmap of modalities used across various disease domains. In oncology, the integration of histopathology images with omics data (e.g., genomics, proteomics) has enabled precision cancer subtyping, while in neurology, combining MRI, PET, EEG, and cognitive assessments has improved early detection of Alzheimer’s disease and seizure classification.

Cardiology and critical care rely on fusing signals such as ECG and vitals with structured EHR data for robust arrhythmia and sepsis diagnosis. Similarly, in pulmonology, multimodal methods integrating chest X-rays or CT scans with radiology reports and clinical data have proven effective in pneumonia and COVID-19 severity assessment. Ophthalmology has seen benefits from fusing retinal imaging with EHR metadata to improve diabetic retinopathy and AMD detection, while dermatology applications, though less mature, show that even limited metadata combined with dermoscopic images can boost melanoma classification accuracy.

Table 13 summarizes representative applications, datasets, modalities, fusion strategies, and challenges across major disease domains. As shown, performance gains are consistent across areas, but limitations such as small datasets, missing modalities, interpretability gaps, and population bias remain critical barriers to clinical translation. These challenges reinforce the need for more comprehensive datasets, robust fusion models, and standardized evaluation protocols in multimodal diagnostic research.

**Table 13:** Applications of multimodal learning in disease diagnosis.

Domain	Key datasets	Modalities used	Fusion strategies	Reported gains	Challenges
Oncology	TCGA, CPTAC	Pathology WSIs + Genomics/Transcriptomics/Proteomics	Intermediate (attention, GNNs); Late fusion ensembles	+5–10% AUROC vs. unimodal	Missing omics data, interpretability
Neurology	ADNI, Epilepsy datasets	MRI + PET + Cognitive scores + Genetics; EEG + MRI	Early fusion; Intermediate fusion (transformers)	Improved early Alzheimer’s detection, seizure classification	Small datasets, longitudinal alignment
Cardiology & Critical Care	MIMIC-III/IV, PhysioNet	ECG, EHR (labs, vitals, notes), Waveforms	Early fusion (concatenation); Late fusion (ensembles)	Higher AUROC in arrhythmia & sepsis detection	Missing labs, real-time integration
Pulmonology	MIMIC-CXR, CheXpert, COVID-CT	Chest X-ray/CT + Radiology Reports + EHR	Early fusion; Vision–language transformers	Significant gains in pneumonia & COVID-19 severity classification	Report variability, population bias
Ophthalmology	UK Biobank	Fundus/OCT + EHR/Demographics	Early fusion; Intermediate transformers	Better sensitivity for DR, AMD diagnosis	Limited public multimodal datasets
Dermatology	ISIC Archive	Dermoscopic images + Metadata (age, sex, lesion site)	Early fusion	Improved melanoma classification	Lacks genomics/longitudinal data

### 9.1 Image–omics and histology–transcriptomics learning

Recent progress in multimodal medical AI has been driven by cross-modal representation learning between histology images and molecular profiles, where the goal is not merely to concatenate features but to learn shared embeddings that align morphology with gene-level or pathway-level states. A representative line of work predicts gene expression directly from whole-slide histology by modeling higher-order tissue relationships using hypergraph neural networks, enabling gene-level inference from cost-effective imaging while capturing structured dependencies among multi-scale morphological features (13). Closely related approaches shift from direct regression toward alignment objectives, using histology-enhanced contrastive learning to impute transcriptomic profiles by explicitly learning morphology–molecular correspondences in a shared space, thereby reducing reliance on expensive spatial transcriptomics assays while preserving biologically meaningful variation (14). Beyond oncology-style histology–omics pairing, multimodal contrastive frameworks have also been applied to genotype–imaging integration for neurodegenerative disease, aligning SNP-derived representations with MRI-derived representations to support both predictive accuracy and mechanistic interpretation in Alzheimer’s disease diagnosis (15). Complementing these alignment-based paradigms, recent work emphasizes trustworthy multimodal mechanisms that explicitly model reliability across modalities for illness classification under heterogeneous data conditions. Finally, cross-modal phenotypic learning for drug discovery demonstrates the broader impact of modern multimodal representation learning, where high-content cellular images are converted into compact, generalizable phenotypic profiles that support robust downstream screening and mechanistic grouping (16). Collectively, these studies exemplify the shift from fusion taxonomies toward representation alignment, structured reasoning, and robustness-aware multimodal learning across image–omics–clinical settings.

As presented in Table 14, the integration of histopathology imaging with transcriptomic and multi-omics data has emerged as one of the most rapidly advancing frontiers in multimodal biomedical AI. Large-scale cancer cohorts such as TCGA and CPTAC provide paired whole-slide images (WSIs) and molecular profiles, enabling direct modeling of the relationship between tissue morphology and gene expression. Unlike traditional multimodal fusion approaches that treat modalities as independent predictive signals, histology–omics learning explicitly seeks to uncover biological correspondence between visual phenotypes and molecular mechanisms.

**Table 14:** Histology–transcriptomics and image–omics multimodal learning.

Study	Modalities	Methodological approach	Dataset
Schmauch et al. (119)	WSI + RNA-seq	Gene expression regression from histology	TCGA
Chen et al. (120)	Histology + multi-omics	Cross-modal alignment with attention	TCGA
Fu et al. (121)	WSI + Genomics	Graph-based multimodal fusion	Oncology cohorts
Ding et al. (122)	WSI + Transcriptomics	Contrastive cross-modal learning	TCGA
Levy-Jurgenson et al. (123)	Histology + RNA-seq	Weakly supervised regression	TCGA
Shao et al. (124)	WSI + Multi-omics	Transformer-based multimodal modeling	TCGA
He et al. (125)	Pathology + Transcriptomics	Graph attention networks	Cancer datasets
Chen et al. (126)	Histology + Omics	Self-supervised multimodal pretraining	Multi-cohort

A central task in this domain is gene expression prediction from histopathology. Formally, given a whole-slide image $m_{\mathrm{WSI}}$ , the objective is to estimate a gene expression vector $g\in {\mathbb{R}}^{d}$ :


$$g^{\prime }=f_{\theta }(m_{\mathrm{WSI}})$$

Where d represents the number of genes or transcripts. The optimization objective is typically formulated as:


$$L=∥g-g\prime {∥}_{2}$$

or through correlation-based metrics to preserve biological ranking consistency. Such approaches demonstrate that tissue morphology encodes rich molecular information, enabling in silico transcriptomics from imaging alone. Beyond direct regression, recent methods adopt cross-modal representation learning frameworks to align WSI embeddings with transcriptomic embeddings. Let $z_{\mathrm{img}}=f(m_{\mathrm{WSI}})$ and $z_{\text{omics}}=h(g)$ ; alignment objectives minimize distance or maximize similarity between modalities:


$${ℒ}_{\text{align}}=-\mathrm{sim}(z_{\mathrm{img}},z_{\text{omics}})$$

This formulation enables shared latent spaces capturing tissue–gene relationships, supporting survival prediction and tumor subtyping tasks.

Graph-based modeling has further enriched histology–omics integration. Tissue regions within WSIs can be treated as spatial nodes, while genes represent molecular nodes in a bipartite or heterogeneous graph. Message passing mechanisms propagate information between spatial and molecular domains, modeling structured tissue–gene interactions:


$$h_{v}^{\left(l+1\right)}=\sigma (W^{\left(l\right)}\sum_{j\in N(v)}W^{\left(l\right)}h_{u}^{\left(l\right)}$$

Such relational modeling captures higher-order dependencies between spatial morphology and transcriptomic signatures. More recently, contrastive learning strategies have been introduced to align pathology and omics embeddings without explicit regression targets. These approaches maximize agreement between matched WSI–omics pairs while minimizing similarity across unmatched pairs, enabling robust representation learning even when gene-level supervision is noisy or incomplete.

## 10 Challenges and open problems

Despite clear performance gains, today’s multimodal diagnostic systems face cross-cutting barriers that limit real-world impact. Data are fragmented and incomplete (patients rarely have every modality), labels can be noisy, and temporal alignment across streams (images, labs, notes, wearables) is inconsistent. Models often overfit to site-specific quirks, struggling to generalize across institutions, scanners, and demographics raising fairness concerns. Powerful joint-fusion architectures are computationally heavy, difficult to deploy for real-time triage, and frequently under-calibrated under distribution shift or missing-modality stress. At the same time, black-box fusion obscures why a system made a decision, complicating clinical trust and accountability. Evaluation practices also lag behind: studies too often report only discrimination (e.g., AUROC) without calibration, decision-curve analysis, or cost-utility, and lack multi-center external validation or prospective testing. Finally, privacy, governance, and consent constraints make it hard to link modalities across silos and to share data for reproducible science. The remainder of this section unpacks these issues—missingness and alignment, data scarcity and bias, generalizability, interpretability and safety, efficiency and deployment, and privacy/ethics—and outlines methodological and infrastructural directions to address them.

### 10.1 Missing modalities and incomplete data

One of the most persistent barriers in multimodal disease diagnosis is the problem of incomplete data availability. In clinical practice, patients rarely undergo the full spectrum of diagnostic tests—an individual may have radiology images but no genetic sequencing, or laboratory values but no corresponding clinical notes. This modality missingness arises due to cost, patient condition, institutional protocols, or logistical constraints. As a result, multimodal models that assume complete data often suffer a significant performance drop when applied in real-world settings. To address this, researchers have explored several strategies. Modality dropout during training simulates missing data scenarios, encouraging robustness at inference time. Generative imputation methods—such as variational autoencoders, Generative Adversarial Network (GANs), or cross-modal transformers—reconstruct missing inputs from available ones, but risk amplifying noise or introducing synthetic bias. Mixture-of-experts (MoE) and late fusion frameworks naturally handle missingness by leveraging whichever modalities are present, though they may fail to exploit deeper cross-modal interactions.

Table 15 highlights representative literature that applied fusion in diagnostic settings under varying assumptions about modality availability. For example, Pathomic Fusio Chen et al. (17) demonstrated strong gains in cancer subtype classification but showed poor robustness when genomic data were absent. Similarly, CsAGP. Tang et al. (18) boosted Alzheimer’s detection using MRI and PET, yet required complete modality pairs for optimal performance. LVH-Fusion Soto et al. (19) and RadFuse Jahanian et al. (20) showcased innovative intermediate fusion approaches but offered limited resilience to missing signals or clinical notes. More robust frameworks, such as Mixture-of-Experts with modality dropout, provide partial solutions but still struggle with calibration and uncertainty estimation. This body of work demonstrates that while partial robustness strategies exist, the challenge of handling incomplete patient data remains unresolved. Progress in this area will require uncertainty-aware fusion, hierarchical architectures that adapt to available modalities, and benchmarking protocols that simulate realistic missingness patterns, ensuring that models align more closely with clinical practice.

**Table 15:** Missing modalities in multimodal diagnosis work so far.

References	Modality setting	Fusion/handling strategy	Limitations
Chen et al. (17)	Histopathology + Genomics	Tensor fusion with gated attention; assumes complete modalities	Not robust to missing omics; performance drops sharply when data incomplete
Tang et al. (18)	MRI + PET (Alzheimer’s)	Cross-attention dual-transformer	Requires full MRI + PET pairs; fails if one modality missing
Soto et al. (19)	ECG + Echocardiogram	Joint time-series modeling	Small dataset; no handling of missing ECG/echo
Jahanian et al. (20)	Chest X-ray + Reports	Vision–language transformer	Radiology notes often missing in practice → model not resilient
Subhalakshmi et al. (81)	CT + Clinical Features (COVID-19)	Early fusion	Imbalanced missing labs; no robust missing-data strategy
Xue et al. (127)	Mixed (EHR, Imaging, Signals)	Mixture-of-experts with modality dropout	Still limited calibration under missingness; hard to train

### 10.2 Data scarcity and imbalance

Another major challenge for multimodal disease diagnosis is the limited availability of large, balanced datasets. While benchmarks such as TCGA, ADNI, and MIMIC have enabled progress, most multimodal datasets are domain-specific, collected from a few centers, and heavily skewed toward common conditions. Rare diseases, pediatric cohorts, and data from low- and middle-income countries remain underrepresented, which undermines the generalizability of models. Furthermore, class imbalance—such as having far more negative cases than positive ones in cancer detection or diabetic retinopathy screening—leads to biased classifiers that may perform well on the majority class but fail in critical clinical settings.

Researchers have proposed several strategies to mitigate scarcity and imbalance. Data augmentation (image transformations, synthetic signal generation) and oversampling techniques such as SMOTE have been applied, though their effectiveness diminishes when modalities need to remain aligned. Generative models (e.g., GANs, Variational Autoencoder (VAEs), diffusion models) can synthesize minority-class samples or missing modalities, but these risk introducing artifacts or biases that diverge from real biological signals. Transfer learning and self-supervised pretraining from large unimodal corpora (e.g., ImageNet for imaging, PubMed for text) are also increasingly popular to bootstrap multimodal systems. Despite these efforts, no approach fully resolves the scarcity problem, as each requires careful validation to ensure synthetic or pre-trained features are clinically reliable.

Table 16 provides an overview of representative studies that have attempted to tackle multimodal data scarcity and imbalance across different disease domains. For example, Liu et al. (21) used transfer learning on ADNI to improve Alzheimer’s detection under small sample sizes, while Han et al. (22) employed cross-modal GANs to augment rare cancer subtypes in TCGA. In the context of COVID-19, Loey et al. (23) balanced minority cases through oversampling, and El-Ateif et al. (24) compared early versus late fusion strategies for ophthalmic diseases under limited samples. Meanwhile, Ngiam et al. (25) demonstrated the promise of self-supervised multimodal pretraining for general healthcare applications. Collectively, these efforts highlight both the creativity of solutions and their limitations: synthetic data may not reflect biological reality, oversampling risks overfitting, and transfer learning often remains dataset-specific.

**Table 16:** Literature on data scarcity and imbalance.

References	Domain/dataset	Strategy used	Limitations
Liu et al. (21)	Neurology (MRI + PET)	Transfer learning + data augmentation	Still dataset-specific; lacks external validation
Han et al. (22)	Oncology (TCGA histology + genomics)	Cross-modal GAN to generate synthetic omics	Synthetic data may not reflect true biology
Loey et al. (23)	Pulmonology (CT + clinical data)	Oversampling minority COVID+ cases	Overfitting on oversampled data
Ngiam et al. (25)	EHR + Imaging	Contrastive learning across modalities	Requires very large pretraining corpora
El-Ateif et al. (24)	Ophthalmology	Comparative early/late fusion under limited samples	Still constrained by small sample bias

### 10.3 Generalizability and fairness

A key barrier to clinical deployment of multimodal models is their limited generalizability across populations, institutions, and acquisition settings. Models trained on single-center datasets often exploit site-specific biases (scanner type, patient demographics, annotation style), resulting in poor performance when evaluated on external cohorts. For instance, chest X-ray datasets like MIMIC-CXR and CheXpert are heavily U.S.-centric, while ophthalmology datasets like UK Biobank are skewed toward European populations. Without exposure to diverse data sources, models may produce unfair or unreliable predictions for underrepresented groups, raising equity and trust concerns in healthcare. Fairness issues also emerge when modalities themselves carry demographic confounders. For example, skin tone variations influence dermatology image models, while EHR data may reflect systemic inequities in access to care. Although domain adaptation, adversarial debiasing, and federated learning approaches have been proposed, ensuring fairness across multimodal inputs remains more complex than in unimodal settings because biases can compound across modalities. Furthermore, most studies report performance only in aggregate (e.g., AUROC) without subgroup analyses, masking disparities.

Table 17 summarizes representative efforts to improve generalizability and fairness in multimodal diagnosis. For example, Rajpurkar et al. (26) evaluated multimodal chest X-ray + report models across multiple hospitals, highlighting site-specific performance drops. Banerjee et al. (27) used federated multimodal learning across oncology centers to improve robustness while preserving privacy. Wu et al. (28) applied adversarial debiasing in dermatology to reduce disparities across skin tones, while Ngiam et al. (25) showed that self-supervised pretraining improved cross-domain transfer for multimodal EHR + imaging. Despite these advances, true fairness requires systematic external validation, diversity in training datasets, and reporting standards that mandate subgroup performance reporting.

**Table 17:** Generalizability and fairness work done so far.

References	Domain/dataset	Strategy used	Limitations
Rajpurkar et al. (26)	Pulmonology (MIMIC-CXR, CheXpert, multi-hospital)	Cross-hospital external validation	Lack of fairness-specific mitigation
Banerjee et al. (27)	Oncology (TCGA + multi-center)	Federated multimodal learning	High communication/computation costs
Wu et al. (28)	Dermatology (ISIC + diverse cohorts)	Adversarial debiasing	Fairness gains limited to image modality
Ngiam et al. (128)	EHR + Imaging (general)	Self-supervised pretraining	Requires very large pretraining corpora
Yang et al. (29)	Neurology (ADNI + external MRI/PET)	Domain adaptation for MRI/PET fusion	Alignment across modalities still imperfect

### 10.4 Interpretability and clinical trust

Beyond raw accuracy, a persistent obstacle for multimodal AI in healthcare is its lack of interpretability. Clinicians require models not only to predict but also to explain the reasoning process, especially when multiple modalities interact in non-obvious ways. In real-world settings, a black-box model that fuses imaging, EHR, and genomic data without clear attribution raises concerns about trust, accountability, and liability. For example, a cancer classifier may combine features from pathology and genomics, but without clarity on which modality contributed most, physicians may hesitate to act on its recommendations. To overcome this, recent work has focused on developing inherently interpretable fusion architectures. Attention-based methods highlight cross-modal weights, while graph-based models represent relationships between modalities in a clinically meaningful way. Other efforts adopt prototype-based reasoning, where predictions are explained by reference to similar patient cases, or causal inference frameworks to separate spurious correlations from clinically relevant signals. Despite progress, interpretability methods often remain limited to specific modalities (e.g., imaging), leaving structured data and omics underexplored. Moreover, many solutions are still post-hoc explanations rather than being embedded in the model design, raising concerns about their reliability.

Table 18 summarizes representative literature in this area. For example, Lal et al. (29) proposed a graph neural network framework to interpret relationships between imaging and omics in cancer studies, while Sun et al. (30) applied prototype-based reasoning to multimodal EHR data, allowing clinicians to trace predictions to similar historical cases. In ophthalmology, Bhardwaj et al. (31) integrated attention heatmaps into multimodal fundus + OCT analysis, improving clinician trust in diabetic retinopathy diagnosis. Similarly, Ghorbani et al. (32) introduced multimodal concept attribution to provide human-understandable explanations in cardiology. More recently, Li et al. (33) developed a causal inference–guided multimodal fusion model for neurodegenerative disease, explicitly disentangling clinically relevant from spurious signals. Collectively, these approaches demonstrate that interpretable multimodal AI is feasible, but remain fragmented, with limited standardization across modalities and diseases.

**Table 18:** The progress of interpretability in multimodal fusion.

References	Domain/dataset	Interpretability strategy	Limitations
Lal et al. (29)	Oncology (multi-omics + pathology)	Graph neural network to model modality relations	Complex, requires large paired datasets
Sun et al. (30)	EHR (multimodal clinical data)	Prototype-based reasoning	May oversimplify complex feature interactions
Bhardwaj et al. (31)	Ophthalmology (fundus + OCT)	Attention heatmaps in multimodal fusion	Heatmaps subjective, not always consistent
Ghorbani et al. (32)	Cardiology (ECG + EHR)	Multimodal concept attribution	Limited scalability beyond ECG/EHR
Li et al. (33)	Neurology (MRI + PET)	Causal inference–guided multimodal fusion	High computational cost; early-stage validation

### 10.5 Computational cost and scalability

While multimodal learning promises richer diagnostic insight, many state-of-the-art fusion models as presenting in Table 19 are computationally demanding, limiting their deployment in clinical practice. Architectures that use cross-attention transformers, 3D Convolutional Neural Network (CNN), or graph networks across modalities require significant GPU resources and are impractical for hospitals with limited computational infrastructure. For example, training multimodal oncology models on histology whole-slide images (WSIs) and omics data can require terabytes of storage and weeks of computation. In ICU and emergency triage, where real-time decision-making is critical, computational latency can make complex fusion approaches unusable.

**Table 19:** Existing literature on computational cost and scalability.

References	Domain/dataset	Efficiency strategy	Limitations
Xu et al. (129)	Oncology (TCGA + WSIs)	Knowledge distillation for multimodal cancer models	Distillation quality depends on teacher model
Kim et al. (130)	Pulmonology (MIMIC-CXR)	Model pruning and quantization for multimodal CXR + reports	Loss of robustness under missing modalities
Albahli et al. (131)	Ophthalmology (fundus + OCT)	Edge deployment of multimodal CNNs	Limited to small-scale models
Chen et al. (132)	Critical Care (MIMIC-IV, EHR + vitals)	Early-exit multimodal transformers	Risk of premature exit on ambiguous cases
Hassan et al. (133)	Dermatology (ISIC + metadata)	Lightweight multimodal fusion with MobileNet backbone	Lower accuracy compared to large transformers

To mitigate these issues, recent works have explored model compression, pruning, and distillation to create lightweight yet accurate multimodal models. Others use early-exit strategies, where predictions can be made at intermediate layers if confidence is high, reducing computation. In resource-constrained settings, edge deployment of multimodal AI has also been investigated, particularly for point-of-care diagnostics in ophthalmology and dermatology. Despite these efforts, achieving a balance between model complexity, interpretability, and speed remains an unresolved problem. Clinical translation requires not only accuracy but also scalability across institutions with diverse hardware environments.

### 10.6 Privacy, ethics, and data sharing

One of the most pressing challenges as presented in Table 20 in multimodal healthcare AI is the sensitive nature of patient data. Multimodal datasets typically combine imaging, laboratory values, genomic sequences, and clinical notes each of which can carry personally identifiable information (PII). Linking these modalities across institutions increases the risk of patient re-identification, even when individual data streams are anonymized. Recent studies have therefore proposed privacy-preserving machine learning frameworks, including secure data sharing, differential privacy, and encrypted computation methods, to protect sensitive medical information while enabling collaborative model training across institutions (34, 35).

**Table 20:** Existing literature on privacy, ethics, and data sharing in multimodal AI.

References	Domain/dataset	Privacy/ethics strategy	Limitations
Rieke et al. (134)	Cross-domain (EHR + imaging)	Federated learning for medical AI	Communication overhead, performance drop vs. centralized models
Sheller et al. (135)	Oncology (BraTS MRI)	Federated learning across hospitals	Still vulnerable to gradient leakage
Kaissis et al. (136)	Multimodal medical imaging	Differential privacy in federated multimodal learning	Reduced accuracy in small datasets
Yan et al. (137)	Cardiology (ECG + EHR)	Blockchain-secured multimodal data sharing	High computational cost, scalability concerns
Wang et al. (135)	Genomics + clinical data	Secure multiparty computation for multi-omics	Limited to small-scale experiments

Technical solutions such as federated learning, differential privacy, and secure multiparty computation have been explored to enable collaboration without raw data sharing. However, these methods often come with trade-offs in accuracy and computational efficiency. Furthermore, ethical concerns extend beyond privacy: biased or unrepresentative multimodal datasets may reinforce health disparities, and the absence of transparent data governance undermines public trust. Without robust frameworks for privacy-preserving training, equitable access, and explainable consent, large-scale multimodal AI systems risk limited clinical adoption.

### 10.7 Methodological synthesis and design principles for multimodal diagnosis

While multimodal disease diagnosis is often presented through application-specific performance gains, the core methodological contribution of the field lies in how models learn cross-modal representations under heterogeneity, incompleteness and distribution shift. Across medical domains, as presented in Table 21, multimodal systems differ less in the diseases they target than in the representation learning assumptions they adopt. From this perspective, multimodal learning can be viewed as the estimation of a latent patient state from heterogeneous observations, where each modality provides a partial and biased projection of the underlying clinical reality. This framing motivates the need to synthesize methodological insights beyond case-wise AUROC reporting.

**Table 21:** Methodological synthesis across modern multimodal paradigms.

Paradigm	How cross-modal representation is learned	Handling modality heterogeneity	Missing-modality strategy	Strength
Classical fusion (early/intermediate/late)	Feature/decision aggregation	Encoder per modality; manual fusion	Weak (assumes full inputs)	Simple, interpretable pipeline
Contrastive alignment	Shared embedding space via similarity maximization	Modality-specific encoders; aligned latent space	Moderate (partial via embedding learning)	Strong transfer learning
Vision–language pretraining	Image–text semantic alignment; transformer grounding	Token-level interaction via attention	Moderate–high (masking tokens)	Scalable, semantically grounded
Graph/hypergraph reasoning	Structured relational embeddings via message passing	Heterogeneity modeled in node/edge types	Moderate (partial graphs possible)	Captures higher-order interactions
Modality-agnostic learning	Invariant patient embedding	Shared latent constraints; adversarial invariance	High (designed for partial inputs)	Flexible across input configurations
Missing-modality robust architectures	Stable prediction under modality subsets	Dynamic gating/weights	High (explicit objective)	Clinically realistic
Foundation multimodal pretraining	Large-scale self-supervised multimodal embeddings	Unified transformer tokenization	High (trained on variable subsets)	Cross-task adaptation

A first unifying theme is how cross-modal representations are learned. Classical fusion strategies treat modalities as independent feature sources and combine them at the feature level or decision level. Modern approaches increasingly learn aligned or shared latent spaces in which heterogeneous modalities encode semantically consistent patient representations. Contrastive alignment and vision–language pretraining represent explicit solutions to this problem by directly optimizing similarity between paired modality embeddings. Graph-based multimodal learning addresses representation learning differently by enforcing relational structure, allowing interactions among imaging regions, clinical variables, and molecular signals to be modeled as structured dependencies rather than implicit correlations. Modality-agnostic learning further generalizes this idea by encouraging invariance to modality identity, effectively learning patient representations that remain stable regardless of which modality is observed.

A second methodological theme concerns modality heterogeneity. Healthcare modalities differ in scale, dimensionality, temporal resolution, missingness patterns, and noise characteristics. Imaging typically provides high-dimensional spatial signals, omics provide high-dimensional molecular profiles with strong sparsity, and EHR data may be irregular, sparse, and institution-specific. Robust multimodal architectures handle this heterogeneity through modality-specific encoders and normalization strategies, attention-based weighting mechanisms, and hierarchical fusion designs that prevent dominant modalities from overwhelming weaker ones. In practice, heterogeneity is not merely a technical inconvenience but a source of systematic bias: modalities are recorded differently across health systems, and documentation practices can imprint spurious correlations into multimodal models. Therefore, methodological robustness requires not only architectural flexibility but also careful evaluation across institutions and patient subgroups.

A third theme concerns missingness as a structural property of real clinical data. Unlike benchmark multimodal datasets, clinical records are incomplete by design; certain modalities are ordered only when clinically indicated, and availability is shaped by resource access and institutional workflows. High-quality multimodal systems therefore require training objectives and architectures that remain stable under partial modality subsets. Mixture-of-experts gating, modality dropout, generative imputation, and uncertainty-aware fusion have emerged as key strategies for addressing missing data, with the most clinically realistic models treating missingness not as noise but as a condition that must be explicitly modeled during learning and evaluation.

Across the literature, these methodological principles reveal that multimodal performance gains are not solely determined by adding modalities, but by how representation alignment, heterogeneity handling, and missingness robustness are jointly addressed. Consequently, beyond reporting improvements in AUROC, a methodologically grounded evaluation should include ablation across modality subsets, external validation across institutions, robustness under missingness simulation, and fairness analyses across population strata. This methodological synthesis clarifies why certain multimodal approaches generalize well while others fail when deployed outside controlled settings, and it provides a more transferable framework for interpreting disease-specific applications presented throughout the survey.

While multimodal healthcare AI faces well-documented challenges including missing modalities, fairness disparities, interpretability limitations, and robustness under distribution shift—recent literature has proposed concrete methodological frameworks to address these issues. Rather than viewing these challenges as abstract limitations, it is important to examine how specific architectural and training strategies attempt to mitigate them. Table 22 provides some of the key solution to this process. As we know that, missing modality remains one of the most fundamental structural challenges in clinical multimodal learning. To address this, mixture-of-experts gating mechanisms dynamically reweight modality-specific encoders based on availability, while modality dropout strategies simulate incomplete inputs during training to enhance robustness. Generative imputation models reconstruct missing modalities through conditional generative networks, and uncertainty-aware fusion frameworks quantify predictive confidence when data are incomplete. These solution families demonstrate that missingness can be modeled as a design constraint rather than treated as noise (144–147).

**Table 22:** Mapping key multimodal challenges to representative methodological solutions.

Challenge	Representative solution frameworks	Example methodological paradigms	Strength
Missing modalities	Mixture-of-experts gating; modality dropout; generative imputation; uncertainty-aware fusion	MoE multimodal models; GAN-based imputation; confidence-calibrated fusion	Improved stability under partial inputs
Fairness disparities	Domain-invariant learning; adversarial debiasing; distributionally robust optimization	Adversarial multimodal encoders; subgroup-aware calibration	Reduced cross-site performance gaps
Interpretability	Graph attention networks; cross-attention visualization; concept bottleneck models	GNN-based multimodal models; vision–language attention maps	Structured explanation of interactions
Distribution shift	Contrastive pretraining; domain generalization; invariant risk minimization	CLIP-style pretraining; IRM-based multimodal training	Better cross-institution generalization
Modality heterogeneity	Modality-specific encoders; hierarchical fusion; invariant embedding constraints	Transformer-based multimodal encoders	Flexible heterogeneous integration

Fairness concerns arise when modality availability or data quality differs across demographic groups or institutions. Domain-invariant representation learning, adversarial debiasing objectives, and distributionally robust optimization have been introduced to mitigate performance disparities. In multimodal systems, fairness-aware weighting mechanisms and subgroup-specific calibration strategies have been shown to reduce predictive bias across race, sex, and socioeconomic strata. However, the integration of fairness constraints into multimodal transformers remains an open research direction. Interpretability challenges are particularly salient in multimodal architectures due to complex cross-modal interactions. Graph attention networks provide structured relational explanations by highlighting influential nodes and edges. Cross-attention heatmaps in vision–language models offer spatial grounding between textual descriptions and image regions. Concept bottleneck models and prototype-based multimodal learning further enhance interpretability by enforcing intermediate human-interpretable representations. Robustness under distribution shift, especially across institutions, has been addressed through contrastive pretraining on diverse datasets, domain generalization techniques, and invariant risk minimization strategies. Large-scale multimodal foundation models also exhibit improved cross-site transfer due to exposure to heterogeneous data distributions during pretraining.

## 11 Future trends

Looking ahead, the field of multimodal learning for disease diagnosis is expected to undergo several transformative shifts that will shape research and clinical adoption in the coming years. One of the most prominent directions is the rise of foundation models. Inspired by advances in large-scale vision-language and clinical models, these architectures are trained on massive multimodal datasets and can be adapted across domains with minimal supervision. Their ability to reduce dependence on labelled data while enabling cross-domain transfer makes them highly promising for medical AI, where annotated datasets are often limited. Closely related is the growing use of self-supervised learning, which leverages pretext tasks such as masking or contrastive learning across imaging, EHR, and physiological signals. By effectively exploiting the vast volumes of unlabelled medical data, self-supervised approaches are expected to yield stronger, more generalizable representations that enhance diagnostic performance.

Another trend that is rapidly gaining traction is federated and privacy-preserving learning. Since healthcare data is distributed across institutions and subject to strict privacy regulations, federated multimodal frameworks supported by homomorphic encryption and differential privacy mechanisms enable secure collaborative model training without the need to centralize sensitive patient data. In parallel, researchers are increasingly recognizing the importance of causal and knowledge-guided fusion. Unlike purely data-driven methods, these approaches incorporate biomedical ontologies, clinical guidelines, and causal graphs to guide model reasoning, which not only improves interpretability but also addresses concerns of bias and fairness, ultimately building clinician trust in multimodal AI.

Finally, the vision of personalized precision medicine is likely to define the long-term future of multimodal diagnosis. By integrating multi-omics data, longitudinal EHR, imaging, and wearable sensor streams, multimodal frameworks will enable continuous monitoring, early detection, and individualized treatment recommendations. This trend will move diagnostic AI beyond static predictions toward dynamic, patient-specific clinical decision support systems. Representative examples of these future directions are summarized in Table 23, which provides a consolidated view of emerging approaches, their anticipated impact, and recent supporting references. Collectively, these trends highlight that the next generation of multimodal AI will not only be more accurate and robust but also more secure, interpretable, and patient-centered, bridging the gap between algorithmic innovation and real-world clinical practice.

**Table 23:** Future trends in multimodal disease diagnosis.

Trend	Example approaches	Expected impact	Recent references
Foundation models	Large-scale multimodal pretraining (vision-language models, clinical foundation models)	Cross-domain transferability, reduced reliance on labeled datasets	Wang et al. (138) and Boecking et al. (92)
Self-supervised learning	Pretext tasks (masking, contrastive learning) across imaging, EHR, and signals	Better utilization of unlabeled data, stronger feature representations	Azizi et al. (95) and Saeed et al. (139)
Federated & privacy-preserving learning	Federated multimodal training, homomorphic encryption, differential privacy	Secure collaboration across institutions, compliance with data governance	Sheller et al. (135) and Xu et al. (129)
Causal & knowledge-guided fusion	Integration of biomedical ontologies, clinical guidelines, causal graphs	Improved interpretability, fairness, and clinician trust	Zhang et al. (140) and Luo et al. (141)
Personalized precision medicine	Patient-specific multimodal integration of omics, longitudinal EHR, imaging, wearables	Early detection, individualized treatment, continuous monitoring	Qiu et al. (142) and Huang et al. (143)

## 12 Conclusion

This survey has explored the evolving landscape of multimodal learning for disease diagnosis, tracing its development from unimodal baselines to sophisticated fusion architectures that integrate imaging, physiological signals, electronic health records, clinical text, and omics data. Through our review of recent literature, datasets, benchmarks, and modeling strategies, it becomes clear that multimodality is not a marginal enhancement but a fundamental necessity for capturing the full spectrum of disease complexity. Unlike unimodal systems, which provide limited and often biased perspectives, multimodal approaches leverage complementary information sources, leading to improved diagnostic accuracy, robustness against missing or noisy data, and enhanced fairness across diverse patient populations. At the same time, the survey has highlighted persistent challenges, including incomplete or missing modalities, data scarcity and imbalance, issues of generalizability and fairness, and concerns around interpretability and clinical trust. These limitations, far from being barriers, point to fertile ground for future research. Promising directions include the adoption of foundation models, self-supervised pretraining, federated and privacy-preserving learning frameworks, knowledge-guided and causal fusion strategies, and the realization of personalized precision medicine through continuous multimodal integration. A summary of these future trends was presented in this survey to provide a roadmap for the next generation of research. In conclusion, the evidence consolidated here demonstrates that multimodal AI holds transformative potential for clinical diagnosis. By aligning methodological innovation with practical clinical needs, the community can move toward diagnostic systems that are not only accurate and scalable but also interpretable, equitable, and trustworthy. The long-term vision is clear: multimodal diagnostic frameworks that function as reliable partners to clinicians, accelerating early detection, refining personalized treatments, and ultimately improving patient outcomes at a global scale.
